# The Role of Bystander Effects in the Antitumor Activity of the Hypoxia-Activated Prodrug PR-104

**DOI:** 10.3389/fonc.2013.00263

**Published:** 2013-10-07

**Authors:** Annika Foehrenbacher, Kashyap Patel, Maria R. Abbattista, Chris P. Guise, Timothy W. Secomb, William R. Wilson, Kevin O. Hicks

**Affiliations:** ^1^Auckland Cancer Society Research Centre, The University of Auckland, Auckland, New Zealand; ^2^Department of Physiology, University of Arizona, Tucson, AZ, USA

**Keywords:** anticancer prodrugs, hypoxia-activated prodrugs, PR-104, bystander effect, extravascular drug transport, multicellular layers, pharmacokinetic/pharmacodynamic modeling, NADPH:cytochrome P450 oxidoreductase

## Abstract

Activation of prodrugs in tumors (e.g., by bioreduction in hypoxic zones) has the potential to generate active metabolites that can diffuse within the tumor microenvironment. Such “bystander effects” may offset spatial heterogeneity in prodrug activation but the relative importance of this effect is not understood. Here, we quantify the contribution of bystander effects to antitumor activity for the first time, by developing a spatially resolved pharmacokinetic/pharmacodynamic (SR-PK/PD) model for PR-104, a phosphate ester pre-prodrug that is converted systemically to the hypoxia-activated prodrug PR-104A. Using Green’s function methods we calculated concentrations of oxygen, PR-104A and its active metabolites, and resultant cell killing, at each point of a mapped three-dimensional tumor microregion. Model parameters were determined *in vitro*, using single cell suspensions to determine relationships between PR-104A metabolism and clonogenic cell killing, and multicellular layer (MCL) cultures to measure tissue diffusion coefficients. LC-MS/MS detection of active metabolites in the extracellular medium following exposure of anoxic single cell suspensions and MCLs to PR-104A confirmed that metabolites can diffuse out of cells and through a tissue-like environment. The SR-PK/PD model estimated that bystander effects contribute 30 and 50% of PR-104 activity in SiHa and HCT116 tumors, respectively. Testing the model by modulating PR-104A-activating reductases and hypoxia in tumor xenografts showed overall clonogenic killing broadly consistent with model predictions. Overall, our data suggest that bystander effects are important in PR-104 antitumor activity, although their reach may be limited by macroregional heterogeneity in hypoxia and reductase expression in tumors. The reported computational and experimental techniques are broadly applicable to all targeted anticancer prodrugs and could be used to identify strategies for rational prodrug optimization.

## Introduction

Intra-tumor heterogeneity is a fundamental barrier to all targeted therapies (1). One of the attractive features of prodrugs that are activated within tumors is their potential for decoupling targeting and pharmacodynamic effect through diffusion of active metabolites from prodrug-activating cells to surrounding untargeted cells. These bystander effects are thought to be important for monotherapy activity of targeted anticancer prodrugs (2–4), including hypoxia-activated prodrugs (HAP) activated by bioreduction in hypoxic regions (5–7). Bystander effects may also be important for activity of HAP in combination with agents that spare hypoxic cells, such as ionizing radiation; activation of most HAP is inhibited by O_2_ concentrations too low to effect radiosensitization (8–11), so there is likely a subpopulation of radioresistant hypoxic cells that can only be killed by HAP if bystander metabolites diffuse from severely hypoxic regions (5). However, the contribution of bystander effects to the anticancer activity of prodrugs, either as monotherapy or in combination settings, is poorly understood.

The purpose of this study was to investigate the role of bystander effects in the antitumor activity of the dinitrobenzamide mustard PR-104, a clinical-stage HAP (12). PR-104 was chosen because its mechanism of action is well understood (7), its active metabolites are known to be capable of diffusing from cells (13) and thus are expected to elicit a bystander effect, and validated analytical methods for their quantitation are available (14). The phosphate ester moiety of PR-104 is rapidly converted systemically to the corresponding alcohol PR-104A (13, 15), which is a prodrug that is activated by reduction of a nitro group to the corresponding hydroxylamine (PR-104H) and amine (PR-104M), both of which are DNA crosslinking cytotoxins (16, 17). Hypoxia-selective activation can be effected by one-electron-reductases such as NADPH:cytochrome P450 oxidoreductase (POR) (18, 19) via formation of a nitro radical that is further reduced to PR-104H and PR-104M under hypoxia, but is rapidly back-oxidized in the presence of O_2_. Half-maximal inhibition of PR-104A cytotoxicity was found to require only ∼0.13 μM O_2_ in SiHa cell suspensions (20), which is well below that for half-maximal radiosensitization [ ∼4 μM O_2_, (21)]. In addition, to this highly O_2_-sensitive one-electron activation mechanism, two-electron reduction by aldo-keto reductase 1C3 (AKR1C3) provides an O_2_-insensitive pathway to the same cytotoxic metabolites in cells with high AKR1C3 expression (22). PR-104 has shown striking single-agent activity in several human tumor xenografts (13, 16, 22, 23), which may partially be due to bystander effects.

Here, we utilize a spatially resolved pharmacokinetic/pharmacodynamic (SR-PK/PD) modeling approach to dissect the contribution of bystander effects (whether from hypoxia-dependent or hypoxia-independent activation) to PR-104 antitumor activity. Our approach builds on the earlier development of an SR-PK/PD model for the well-studied HAP tirapazamine that used Green’s function methods to model diffusion and reaction of O_2_ and tirapazamine in a mapped three-dimensional tumor microregion (24). This model, validated using a series of tirapazamine analogs, demonstrated that rapid bioreductive metabolism during diffusion into hypoxic regions can limit hypoxic cell killing. This led us to use SR-PK/PD modeling to identify tirapazamine analogs with improved extravascular transport and antitumor activity in xenograft (25). The SR-PK/PD models for tirapazamine analogs did not require inclusion of bystander effects, consistent with evidence that the active metabolites are free radicals that do not escape the cell of origin (26). An analogous SR-PK/PD model for PR-104 under-predicted activity in SiHa tumor xenografts, which we suggested might reflect the failure to consider bystander effects (20).

**Figure 1:**
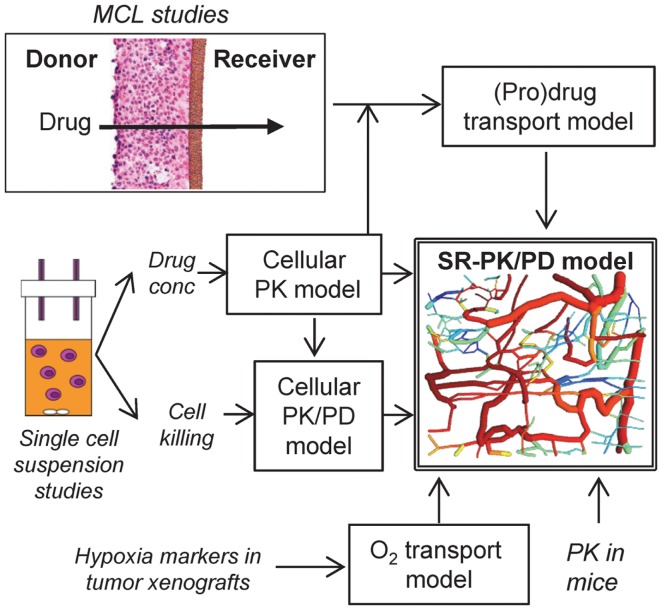
**Approach for the development of an SR-PK/PD model**. The SR-PK/PD model incorporates several different sub-models to calculate O_2_, prodrug and drug AUC (PK) and killing (PD) in a 3D tumor microregion. The cellular PK model describes intra- and extracellular concentration-time profiles of prodrug and drug measured in single cell suspensions. The cellular PK/PD model defines the relationship between cell kill and intracellular drug AUC based on single cell suspension data. Transport of prodrug and drug through MCLs was measured to build a (pro)drug transport model that calculates tissue transport using diffusion coefficients and the parameters of the cellular PK model. The O_2_ transport model, which is informed by the measured binding of 2-nitroimidazole hypoxia markers in tumor xenografts, calculates O_2_ concentrations in the 3D tumor microregion. The (pro)drug transport model then calculates AUC of prodrug and drug at each point of the microregion, using measured PK in mice to define inflow to the network. This information is used by the cellular PK/PD model to estimate cell kill at each position.

In the present study, we develop a PR-104 SR-PK/PD model that explicitly considers bystander effects for the first time, by incorporating reaction and diffusion of the active metabolites of PR-104A (Figure 1). Parameters of the model are determined experimentally using single cell suspensions to develop a cellular PK/PD model that defines relationships between PR-104A metabolism and reproductive cell death (measured as clonogenic cell killing), and multicellular layer (MCL) cultures (27) to determine extravascular transport properties of PR-104A, PR-104H, and PR-10M. These parameters are used to calculate the spatial distribution of PR-104A and its active metabolites, and resulting cell killing, in a virtual tumor microregion that is based on an experimentally observed vascular network structure in a FaDu tumor. This relatively complex approach was used because simpler models assuming regularly spaced vascular geometries under-estimate the spatial heterogeneity of tumor oxygenation (28). Bystander killing is expected to be sensitive to the spatial O_2_ distribution because it critically depends on the distance between severely hypoxic PR-104A-activating cells and bystander target cells at intermediate O_2_ concentrations. We evaluate the SR-PK/PD model by testing its ability to predict measured PR-104 activity in different tumor xenograft models and utilize it to investigate the relative importance of bystander effects in PR-104 antitumor activity.

## Materials and Methods

### The PR-104 SR-PK/PD model

Tissue gradients of oxygen, PR-104A, PR-104H, and PR-104M were calculated in a digitized three-dimensional (3D) tumor microvascular network using Green’s function methods (24, 29). The network was derived by mapping microvascular anatomy as well as direction and velocity of blood flow in a region of a subcutaneous FaDu tumor xenograft (990 μm × 810 μm × 150 μm) grown in a mouse dorsal window chamber (30), and is represented by cylindrical segments (see Figure 1). The vessel walls are treated as part of the tissue space, which is represented as a homogeneous medium. Steady-state conditions are assumed. The model was implemented using a customized version of the Green’s function method written in Visual C++ (Microsoft Visual Studio 2010 Express).

#### Calculation of oxygenation in the tumor microregion

Convective transport of oxygen along vessel segments and diffusion into the surrounding tissue was calculated based on estimates for blood content, tissue diffusion and consumption of O_2_ (29). The O_2_ content of inflowing blood was adjusted to achieve a hypoxic fraction in the tumor microregion that is similar to the measured fraction of HCT116 or SiHa tumor xenografts staining positive for the 2-nitroimidazole hypoxia probe pimonidazole (22) or EF5 (this study). For this purpose a threshold of 1 μM O_2_ was chosen based on the reported O_2_-dependence of 2-nitroimidazole binding (31, 32). O_2_ transport parameters are given in Table S1 in Supplementary Material.

#### Calculation of pharmacokinetics in the tumor microregion

Inflow of PR-104A, PR-104H, and PR-104M to the tumor microvascular network was defined by the plasma pharmacokinetics measured after administration of 562 μmol/kg PR-104 to CD-1 nude (13, 33) or NIH-III nude mice (this study). The active metabolites are present in plasma due to activation of PR-104A in the liver (33). Unbound area under the concentration-time curve (AUC) was used as a time-independent exposure variable compatible with Green’s function formalism.

**Figure 2:**
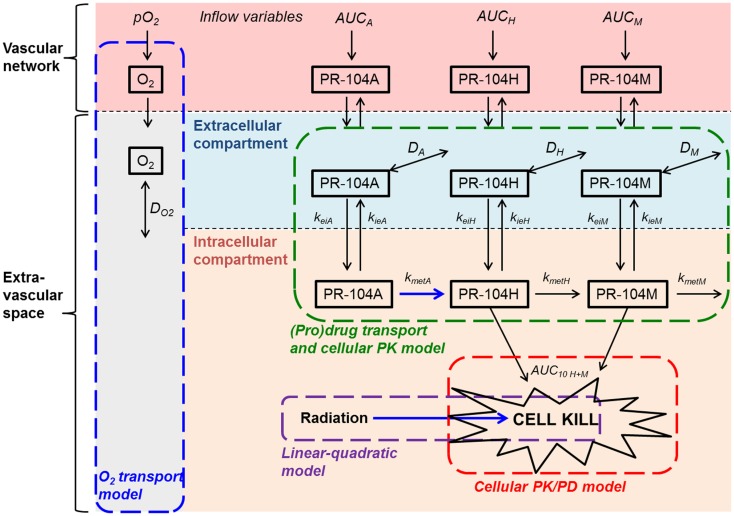
**Schematic representation of the cellular PK/PD and (pro)drug transport models**. The (pro)drug transport model describes PR-104A, PR-104H, and PR-104M concentrations in the extracellular and intracellular compartments. Transfer between the compartments is defined by rate constants *k*_*ei*_*_N_* and *k*_*ie*_*_N_*, where *N* refers to each compound. Loss of PR-104A, PR-104H, and PR-104M by non-enzymatic processes (defined by *k*_instab_*_N_*; not shown) is assumed to occur in both compartments, while drug metabolism (defined by *k*_met_*_N_*) is restricted to the intracellular compartment. In the extracellular compartment compounds can diffuse as defined by their diffusion coefficients *D_N_* (double-headed arrows). The cellular PK/PD model determines cell kill resulting from exposure to PR-104H plus PR-104M (Eq. 4) while the linear-quadratic model calculates radiation-induced killing. The O_2_ transport model calculates convective transport of oxygen along vessel segments and diffusion into the surrounding tissue as described (29) and does not distinguish extra- and intracellular compartments. Arrows in blue indicate O_2_-dependent processes.

Based on the reported high permeability of tumor blood vessels (34, 35), vessel walls were modeled as offering negligible resistance to radial flux of PR-104A, PR-104H, and PR-104M (by setting the intravascular resistance constant (29) to a low value of 0.1 s/μm). Extravascular transport in the tumor tissue was calculated using a 2-compartment (pro)drug transport model (Figure 2) with concentrations in the extracellular compartment (Eq. 1) and the intracellular compartment (Eq. 2) calculated as follows:

(1) $$\begin{array}{l} \varphi_{e}\frac{\partial C_{eN}}{\partial t}=D_{N}\nabla^{2}C_{eN}-\varphi_{i}\left(k_{eiN}C_{eN}-k_{ieN}C_{iN}\right)\\ \;-\varphi_{e}k_{\text{instab}N}C_{eN} \end{array}$$

(2) $$\begin{array}{l} \varphi_{i}\frac{\partial C_{iN}}{\partial t}=\varphi_{i}\left(k_{eiN}C_{eN}-k_{ieN}C_{iN}\right)-\varphi_{\text{i}}k_{\text{instab}N}C_{iN}\\ \;-\varphi_{i}k_{\text{met}N}C_{iN}+r_{N} \end{array}$$

*C*_*e*_ and *C*_*i*_ are the extracellular and intracellular concentrations, respectively, of PR-104A, PR-104H, or PR-104M (denoted by *N* = *A*, *H*, or *M*), φ_*i*_ and φ_*e*_ are the intra- and extracellular volume fractions with φ_*e*_ = 1 − φ_*i*_, *k*_*ie*_*_N_* and *k*_*ei*_*_N_* are the rate constants for transfer from the intracellular to the extracellular compartment and vice versa, *D_N_* is the diffusion coefficient in the extracellular compartment, ▽^2^ is the Laplacian operator, *k*_met_*_N_* and *k*_instab_*_N_* are the rate constants for metabolism and instability, respectively, and *r_N_* is the rate of metabolic production of PR-104H from PR-104A or PR-104M from PR-104H. The rate constant for PR-104A metabolism, *k*_met_*_A_*, is O_2_-dependent:

(3) $$\begin{array}{l} k_{\text{met}A}=f\left[\left({\text{O}}_{2}\right)\right]k_{\text{met}A,\max}\\ \;=\left(\frac{k_{\text{met}A,\min}}{k_{\text{met}A,\max}}+\left(1-\frac{k_{\text{met}A,\min}}{k_{\text{met}A,\max}}\right)\frac{K_{{\text{O}}_{2}}}{K_{{\text{O}}_{2}}+\left[{\text{O}}_{2}\right]}\right)k_{\text{met}A,\max} \end{array}$$

where *f* ([O_2_]) is the ratio of prodrug activation at O_2_ concentration [O_2_] to that under anoxia, $K_{{\text{O}}_{2}}$ is the O_2_ concentration for half-maximum PR-104A activation and *k*_met_*_A_*_,max_ and *k*_met_*_A_*_,min_ are the maximum (anoxic) and minimum (aerobic) rate constants for PR-104A metabolism.

#### Calculation of cell killing in the tumor microregion

Surviving fraction (SF) at each point of the tumor microregion was calculated from intracellular PR-104H + M AUC (*AUC_H_*_+_*_M_*) to account for bystander effects resulting from metabolite diffusion (“+bystander model”; Eq. 4), but from intracellular PR-104A AUC (*AUC_A_*) when assuming that only prodrug-activating cells are killed (“no-bystander model”; Eq. 5). For the former, PR-104H and PR-104M were assumed to be equally potent, based on their similar inhibition of proliferation of several cell lines (17).

(4) $$\text{Log cell kill}=-\log\;SF=\frac{AUC_{H+M}}{AUC_{10H+M}}$$

(5) $$\text{Log cell kill}=f\left(\left[{\text{O}}_{\text{2}}\right]\right)\frac{AUC_{A}}{AUC_{10\;A}}$$

*AUC*_10_*_A_* and *AUC*_10_*_H_*_+_*_M_* are the values of *AUC_A_* (under anoxia) and *AUC_H_*_+_*_M_* (O_2_-independent), respectively, for 10% SF, and *f*([O_2_]) (see Eq. 3) defines the O_2_-dependence of PR-104A cytotoxicity.

Cell survival after radiation treatment was calculated as described (24). Briefly, a linear-quadratic (LQ) model was used:

(6) $$-\log\;SF=\alpha_{H}OER_{\alpha }D_{r}+\beta_{H}{\left(OER_{\beta }D_{r}\right)}^{2}$$

where SF is the cell SF following radiation treatment, *D*_r_ the radiation dose, and α*_H_* and β*_H_* are the proportionality constants for the LQ model under hypoxia. The O_2_ enhancement ratio OER (radiation dose under hypoxia divided by the dose for the same effect at a given oxygen concentration [O_2_]) is calculated by:

(7) $$OER_{i}=\frac{OER_{i,\max}\left[{\text{O}}_{\text{2}}\right]+K_{\text{ms}}}{\left[{\text{O}}_{\text{2}}\right]+K_{\text{ms}}}$$

where *i* denotes α or β, OER*_i_*_,max_ is the maximal O_2_ enhancement ratio and *K*_ms_ the O_2_ concentration for half-maximal radiosensitivity.

The SF from both radiation and prodrug at each point of the tumor microregion was calculated from the sum of log cell kill due to drug and radiation alone.

Averaging SF over the whole tumor microregion gave the overall SF that was used for calculation of overall log cell kill. PR-104-induced cell kill in addition to radiation was calculated as the difference between overall log cell kill by PR-104+ radiation and log cell kill by radiation alone.

### Compound formulation

PR-104 and its metabolites (PR-104A, PR-104H, and PR-10M), and their stable isotope internal standards were synthesized as described (33). EF5 was a gift from the National Cancer Institute (Bethesda, MD, USA). FSL-61 was synthesized as reported (36). All compounds had a purity of >90% by HPLC except PR-104M (86%) and PR-104M-d4 (84%). *In vitro* experiments used frozen (−80°C) stock solutions in DMSO or acetonitrile, diluted at least 100-fold in culture medium. For *in vivo* studies PR-104 was formulated as described (13).

### Cell culture

Origins and monolayer culture of parental cell lines are described elsewhere (19). The expression vector F279-V5 [constructed from pIRES-P (37) and pcDNA6.2V5DEST (Invitrogen)] containing a soluble version of the human POR gene (lacking the first 180 bp encoding the N-terminal endoplasmic reticulum membrane anchor) was prepared by Gateway cloning and used to transfect HCT116/wild type (WT) cells using reported methods (22). The stable clonal cell line HCT116/sPOR#6 was selected with puromycin as described (38) and grown in the presence of 3 μM puromycin. Multicellular spheroids were initiated by seeding 10^5^ cells in bacteriological 100 mm dishes, grown for 3 days, then transferred to spinner flasks and grown for an additional 7 days in αMEM containing 10% FBS and 1% penicillin/streptomycin. Spheroids were enzymatically dissociated by incubation with 0.25% trypsin/EDTA in saline for 10–15 min, followed by incubation with 0.1 mg/ml DNase for 2–10 min. MCLs were grown by seeding 10^6^ cells on microporous support membranes as described (39).

### Cellular PK/PD studies

Intra/extracellular partitioning, (pro)drug metabolism and cytotoxicity were assessed in single cell suspensions as described (40). Briefly, single cells in αMEM without serum (10 ml at ∼2 × 10^6^ cells/ml) were magnetically stirred in glass vials gassed with 5% CO_2_/95% N_2_ (<10 ppm O_2_) or 5% CO_2_/95% air (20% O_2_). Following equilibration for 1 h, (pro)drug [or DMSO alone to determine control plating efficiency (PE)] was introduced and 1 ml samples were transferred to ice-cold glass vials at intervals. Samples were used to evaluate clonogenic cell survival, and extra- and intracellular drug concentrations.

For the latter, cells and medium were separated by centrifugation (12,000 *g*, 30 s) followed by a brief second spin (12,000 *g*, 15 s) to remove excess media. Extracellular samples (supernatants extracted with one volume of cold acidified methanol (methanol:ammonium acetate:acetic acid 1000:3.5:0.2, v/w/v) containing 1 μM PR-104A-d4 and 0.3 μM of PR-104H-d4 and PR-104M-d4) and extracted cell pellets (80 μl of the above extraction solvent per pellet, vortex mixed for 30 s) were frozen at −80°C. Subsequently, thawed cell extracts were centrifuged (13,000 *g*, 4°C, 5 min) and supernatants were diluted in an equal volume of cold αMEM to prepare intracellular samples, which were stored at −80°C until LC-MS/MS analysis. To correct for the contribution from extracellular medium in cell pellets, the cell-excluded marker ^3^H-mannitol was used. Cell suspensions were transferred to microcentrifuge tubes containing 1% (v/v) of 10 pM ^3^H-mannitol (20 *C*_i_/mmol; American Radiolabeled Chemicals Inc., USA). Extracellular and intracellular samples were prepared as above and 25 μl aliquots were mixed with 3 ml of Emulsifier-Safe^TM^ water-accepting scintillant (3 ml; PerkinElmer, Torrance, CA, USA) for scintillation counting (Packard Tricarb Scintillation Analyzer). The fraction of extracellular water in the cell extract (α) can be calculated from the ^3^H-mannitol counts per microliter of the intracellular and extracellular samples:

(8) $$\begin{array}{l} \alpha =\frac{{\;}^{\text{3}}\text{H}-\text{mannitol in intracellular sample}}{{\;}^{\text{3}}\text{H}-\text{mannitol in extracellular sample}}\\ \;=\frac{V_{\text{ec}}}{V_{\text{t}}}=\frac{V_{\text{ec}}}{V_{\text{sol}}+V_{\text{ec}}+V_{\text{c}}} \end{array}$$

with the total volume *V*_t_ of the cell extract being composed of the volume of extraction solvent *V*_sol_, the volume of extracellular water *V*_ec_, and the cellular volume of the cell pellet *V*_c_. The latter was derived by multiplying median cell volume by the cell number (both parameters determined using an electronic cell counter (Z2 Coulter Counter; Beckman Coulter™, USA). The contribution of *V*_ec_ and *V*_c_ is minor, together representing ∼ 4% of *V*_t_. Intracellular concentrations of analytes were estimated by using *V*_ec_ to subtract the contribution of extracellular analytes from the total analytes measured in cell pellet extracts.

Area under the concentration-time curves were calculated from concentration-time profiles using the trapezoidal rule. The relationship between tumor cell survival and drug or prodrug AUC was determined by fitting Eqs 4 or 5 [with *f* ([O_2_]) = 1] to the data using Microsoft Excel 2007.

### MCL studies

Multicellular layers were placed between a donor and a receiver compartment (Figure 1) and equilibrated for at least 1 h under flowing 5% CO_2_/95% O_2_ (oxia) or 5% CO_2_/95% N_2_ (anoxia). PR-104A or PR-104H was added to the donor compartment along with ∼0.4 μM ^14^C-urea (2.183 GBq/mmol; Amersham, Australia) to determine MCL thickness using the known diffusion coefficient of ^14^C-urea in SiHa (41) and HCT116 (39) MCLs. At intervals, 100 μl was sampled from each compartment for liquid scintillation counting, and for LC-MS/MS analysis of PR-104A and metabolites. For the latter samples were processed as described in Section “Cellular PK/PD Studies” for extracellular samples from cellular PK/PD studies.

### Parameter estimation

All model parameters were determined by fitting Eqs 1 and 2 (without including spatial variation) to the concentration-time profiles of PR-104A, PR-104H, and PR-104M measured in cellular PK/PD and MCL studies.

Values of *k*_met_*_N_*, *k*_*ie*_*_N_*, and *k*_*ei*_*_N_* for PR-104A, PR-104H, and PR-104M were simultaneously fitted to cellular PK data, with *D* set to 0, φ_*i*_ set to the cell volume fraction in single cell suspensions (calculated from median cell volume and cell density as measured with an electronic cell counter in each experiment) and *k*_instab_*_N_* fixed at the values determined after addition of the respective compound to culture medium without cells. Non-linear mixed effects modeling (Non-MEM version 7, ICON Development Solutions) using first-order conditional estimation with interaction and ADVAN13 to solve the differential equations was used, allowing for variability of parameters to account for intra- and inter-experiment variability.

Drug transport in MCLs was modeled as one-dimensional diffusion with reaction in the series of compartments (donor, MCL, support membrane, and receiver) using a custom designed MatLab routine. Donor and receiver compartments were modeled as continuously stirred by using a high value for the diffusion coefficient. Diffusion in the support membrane was defined by the effective volume-averaged diffusion coefficients *D*_sup_ of 7.67 × 10^−7^ cm^2^/s (PR-104A), 1.60 × 10^−6^ cm^2^/s (PR-104H), and 6.95 × 10^−7^ cm^2^/s (PR-104M) fitted to support membrane transport data as reported (39). A one-dimensional time-dependent solution of Eq. 1 was fitted to the MCL transport data with *D* and φ_*i*_ as fitted parameters, using a MatLab non-linear regression routine, nlinfit, with 100-fold weighting of receiver compartment data. Here *k*_met_*_N_*, *k*_*ie*_*_N_*, and *k*_*ei*_*_N_* were set at their population mean values (i.e., inter- and intra-experimental variability was not included in the SR-PK/PD model) and parameters estimated in an iterative process until values were found that described both *in vitro* models.

### Tumor models and treatment

All animal studies were approved by the University of Auckland Animal Ethics Committee. Human tumor xenografts were grown subcutaneously on the right flank of female NIH-III nude mice (NIH-Lyst^bg^Foxn1^nu^Btk^xid^; 18–20 g body weight), derived from breeding mice purchased from Charles River Laboratories (Wilmington, MA, USA), by inoculating 0.5–1 × 10^7^ cells. Mice were stratified to treatment groups when tumors reached volumes of 400–800 mm^3^. Compounds were administered by intraperitoneal (i.p.) injection (dose volumes: 0.01–0.02 ml/g). Whole-body irradiation (0.35 Gy/min) was performed using a ^60^Co source. For modulation of tumor oxygenation, animals breathed 100% O_2_ at 2.25 atm (hyperbaric oxygen) in a Reimers RSI B11 hyperbaric chamber (Reimers Systems, USA) or 10% O_2_/90% N_2_ at atmospheric pressure (42). Tumors were excised and single cells prepared by mincing, incubation with enzyme cocktail (2.5 mg/ml pronase, 1 mg/ml collagenase, and 0.2 mg/ml DNAase I) for 30 min at 37°C and sequential filtration using 100, 70, and 40 μm cell strainers (BD Biosciences, USA). For pharmacokinetic studies, plasma and tissue was collected and prepared as described (33) with minor changes: plasma and tissue samples were stored at −80°C before extraction with cold acidified methanol (as above) containing 0.67 μM PR-104-d4, 0.67 μM PR-104A-d4, 0.2 μM PR-104H-d4, and 0.2 μM PR-104M-d4. Extracts were stored at −80°C, centrifuged (13,000 g, 4°C, 10 min) and supernatants were diluted 1:1 with cold water prior to LC-MS/MS analysis.

### Clonogenic assay

Single cell suspensions were serially diluted and plated in 5 ml α-MEM + 5% FBS + 1% penicillin/streptomycin in 60 mm cell culture dishes. Following incubation at 37°C for 11 (HCT116 *in vitro* samples), 12 (HCT116 tumor samples), or 14 (SiHa samples) days, dishes were stained with methylene blue and colonies (>50 cells) were counted to determine PE. Cell SF was determined as PE (treated)/PE (controls). HCT116/sPOR#6 control cells were plated in media with and without 3 μM puromycin to determine the proportion of cells retaining puromycin resistance.

### LC-MS/MS analysis of PR-104 and metabolites

High pressure liquid chromatography with mass spectrometry detection (LC-MS/MS) of PR-104 and metabolites was performed using a validated method (14), with the following changes: the LC-MS/MS system was an Agilent 1100 HPLC interfaced with an Agilent 6410 triple quadrupole mass spectrometer equipped with a multimode ionization source (Agilent Technologies, USA). Chromatographic separation was achieved on a Zorbax SB-C18 rapid resolution column (50 mm × 3 mm, 1.8 μm particles; Agilent Technologies) at 25°C with a 0.2 μm in-line filter. The mobile phase consisted of acetonitrile (A) and 0.01% formic acid in water with fast gradient elution at a flow rate of 0.4 ml/min and run time of 7 min. The following gradient was applied: 0 min, 20% A; 1 min, 20% A; 3.5 min, 40% A; 4 min, 100% A; 5 min, 100% A; 5.5 min, 20% A. The eluent flow was led into the mass spectrometer starting 2 min after injection by switching to the MS inlet valve. PR-104A absorbance was monitored by photodiode array detection upstream of the MS/MS at 370 nm (bandwidth 4 nm, reference wavelength 550 nm) and used for PR-104A quantification in *in vitro* samples with >10 μM PR-104A. Multiple reaction monitoring was used for quantification of PR-104A in *in vitro* samples with <10 μM PR-104A and *in vivo* samples, and for quantification of PR-104, PR-104H, and PR-104M.

Calibration curves were prepared by spiking cold αMEM (*in vitro* studies), blank plasma, or blank tissue extracts (*in vivo* studies) with PR-104A, PR-104H, PR-104M, and PR-104 (only for *in vivo* studies), followed by serial dilution in the respective matrix. The samples were mixed 1:1 with water (tissue extracts) or with the same solvent that was used for sample extraction (*in vitro* and plasma samples) and stored at −80°C.

### Flow cytometry

Reduction of FSL-61 by one-electron-reductases was assessed by flow cytometry as described (43). Staining of EF5 adducts for flow cytometry was performed using Cy-5-conjugated Elk3-51 antibody (Prof. CJ Koch, Pennsylvania University; 75 μg/ml) according to a validated protocol (44). Gates for EF5-positive cells excluded ≥95% of cells from tumors not treated with EF5 but stained with Elk3-51.

### Immunohistochemistry and microscopy

Formalin-fixed paraffin-embedded tumor sections were prepared for immunohistochemistry as reported (42). POR immunostaining was performed using a validated method (19) as described (42). For dual staining of EF5 and pimonidazole, sections were incubated with Cy-3-conjugated Elk3-51 antibody (Pennsylvania University; 100 μg/ml) for 5 h at 4°C, followed by rinsing in Tris buffered saline (pH 7.6) containing 0.1% Tween-20, and incubation with FITC-conjugated anti-pimonidazole antibody (Hypoxyprobe-1 clone 4.3.11.3, Natural Pharmacia International, USA; 120 μg/ml) for 2 h at room temperature.

For imaging of tumor hypoxia, vasculature, and perfusion, tumor-bearing male NIH-III nude mice were dosed i.p. with 60 mg/kg EF5. Three hours later, mice were dosed i.v. with 15 mg/kg Hoechst 33342 (Sigma-Aldrich, USA) and sacrificed 2 min later. Tumors were excised, frozen to liquid nitrogen temperature in Tissue-Tek CRYO-OCT compound (Thermo Fisher Scientific), and stored at −80°C. 10 μm sections were cut using a cryotome, mounted onto glass slides, and stored at −20°C. Following imaging of Hoechst 33342 as below, sections were fixed in ice-cold acetone for 10 min, blocked with 10% normal goat serum in PBS (1 h, RT), and incubated with Alexa Fluor 488-labeled rat monoclonal anti-mouse CD31 antibody (BioLegend, San Diego, CA, USA; 5 μg/ml) overnight at 4°C. After rinsing in PBS, sections were incubated with Cy-3-conjugated Elk3-51 antibody (75 μg/ml) for 5 h at 4°C. Slides were then washed in PBS and counter-stained with 0.5 μg/ml DAPI (Invitrogen Molecular Probes, USA). Whole-section montage images were acquired with a Nikon TE2000E inverted microscope with a 10× objective, using a computer-controlled automatic stage (ProscanII; Prior, USA) and the image acquisition software Image Pro Plus (version 7.1; MediaCybernetics).

## Results

### Development of SR-PK/PD models for three cell lines

Using the approach illustrated in Figure 1, SR-PK/PD models were developed for SiHa and HCT116 cells, which have high and low expression of the aerobic PR-104A reductase AKR1C3 respectively (22), and for HCT116/sPOR#6 cells engineered to provide a high rate of PR-104A activation under hypoxia. Notably, the parental cell lines lack connexin 43 expression (45, 46), thus minimizing any gap junction-dependent drug diffusion.

#### The cellular PK model

**Figure 3:**
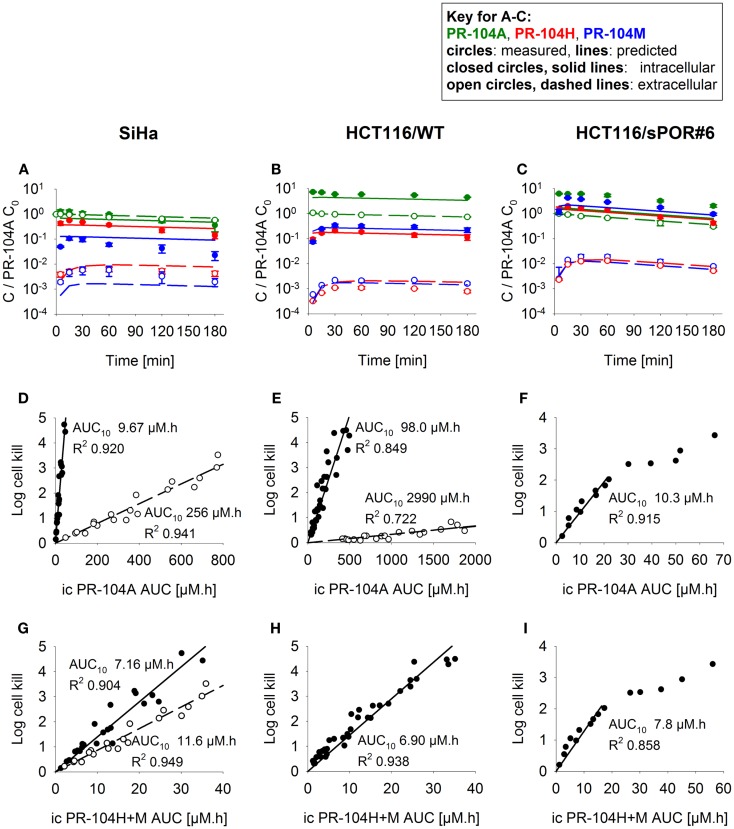
**PK/PD of PR-104A, PR-104H, and PR-104M in single cell suspensions (2 × 10^6^ cells/ml) after addition of PR-104A**. Cells were derived from SiHa monolayers **(A,D,G)** or from spheroids grown from HCT116/WT **(B,E,H)** or HCT116/sPOR#6 cells **(C,F,I)**. **(A–C)** Concentrations of intracellular (closed circles, solid lines) and extracellular (open circles, dashed lines) PR-104A (green), PR-104H (red), and PR-104M (blue) under anoxia. Symbols represent mean ± SEM of concentrations for 4 **(B)** or 3 **(A,C)** replicate vials and lines show predictions of the model that best fitted the experiments overall (including MCL experiments, see Figure 5). Concentrations (*y*-axis) are normalized to initial extracellular PR-104A concentrations (*C*_0_) of 10, 60, and 300 μM **(A)**, 30 μM (*n* = 2) and 40 μM (*n* = 2) **(B)** or 2, 3, and 5 μM **(C)**. **(D–I)** Measured log cell kill under anoxic (●) or aerobic conditions (○) as a function of intracellular (ic) PR-104A AUC **(D–F)** or PR-104H + M AUC **(G–I)**. Symbols represent surviving fractions from individual vials at different time points while lines show linear regression fits. PR-104H + M PK/PD in HCT116/WT and sPOR#6 **(H,I)** is only shown for anoxic conditions because reduced metabolite concentrations were below the LLOQ under aerobic conditions. In SiHa single cell suspensions, intracellular concentrations and cell kill were not measured in the same experiments, therefore intracellular AUC values were derived from model-estimated instead of measured concentration-time profiles.

Firstly the cellular pharmacology of PR-104A was investigated in single cell suspensions. This showed rapid uptake of PR-104A into all cell types (Figures 3A–C). The steady-state *C*_*i*_/*C*_*e*_ ratio was ∼1 in SiHa but >1 in the HCT116 cell lines (range 3–7). Under anoxia PR-104A was converted to the active metabolites PR-104H and PR-104M, which reached much higher concentrations within cells than in the extracellular medium with the following *C*_i_/*C*_e_ ratios at steady-state (30–180 min): SiHa: 56 ± 7 (H), 19 ± 4 (M); HCT116/WT: 160 ± 9 (H), 140 ± 6 (M); HCT116/sPOR#6: 100 ± 8 (H), 160 ± 10 (M). This indicates that cell membranes may act as a barrier to the diffusion of PR-104H + M after their intracellular formation from PR-104A, which could retard their transport in tissue. We therefore distinguished the intracellular and extracellular compartments in the SR-PK/PD model by using a continuum approximation in which transfer between the two compartments is represented by rate constants *k*_*ei*_*_N_* and *k*_*ie*_*_N_*, and it is assumed that metabolism is restricted to the intracellular compartment while diffusion is confined to the extracellular compartment (Figure 2). This cellular PK model contains multiple parameters, which were constrained by the requirement that they also describe MCL transport data (below). For each cell line, parameter sets could be found (Table S1 in Supplementary Material) that globally fitted the data (shown for single cell suspensions in Figures 3A–C). Metabolism of PR-104A, PR-104H, and PR-104M was assumed to follow first-order kinetics because no major non-linearity was found in cellular PK experiments. The cellular PK of HCT116/sPOR#6 cells could be described using the same model as for HCT116/WT apart from a 20.4-fold higher anoxic PR-104A metabolism rate constant (*k*_met_*_A_*_,max_; 1.8 × 10^−2^ s^−1^; Figure 3C), consistent with overexpression of this known (17) PR-104A one-electron-reductase.

#### The cellular PK/PD model

**Figure 4:**
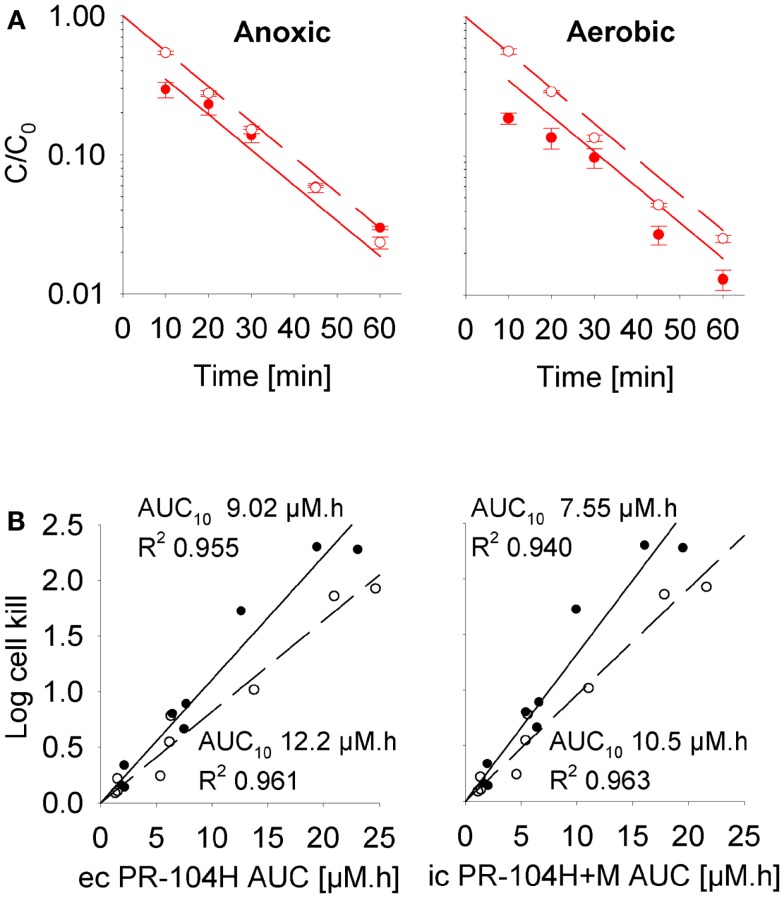
**Cellular PK/PD of PR-104H in SiHa cells**. **(A)** Concentration-time profiles of intracellular (closed circles, solid lines) and extracellular (open circles, dashed lines) PR-104H in SiHa single cell suspensions (5 × 10^6^ cells/ml) following addition of PR-104H under anoxic or aerobic conditions. Concentrations are normalized to initial concentrations (*C*_0_) of PR-104H. All symbols represent mean ± SEM of measured values for three vials (with *C*_0_ of 10, 30, and 100 μM) while lines show predictions of the cellular PK model (see Table S1 in Supplementary Material). **(B)** Measured log cell kill under anoxic (∙) or aerobic conditions (∘) in the above experiment as a function of extracellular (ec) PR-104H AUC as calculated from measured concentration-time profiles shown in **(A)** (left panel), or intracellular (ic) PR-104H + M AUC derived from model-estimated concentration-time profiles (right panel). Lines show linear regression fits.

To define a cellular PK/PD model, we measured clonogenic cell killing as the PD endpoint because this is equally applicable to cell cultures and tumor xenografts. Log cell kill in single cell suspensions was linearly dependent on the AUC of PR-104A or its reduced metabolites (Figures 3D–I), consistent with the linear relationship between PD and AUC for other alkylating agents (47). Data for anoxic HCT116/sPOR#6 cells at low survival (>2 log cell kill) deviated from the linear trend (Figure 3F), which may reflect the presence of a small fraction of cells with low sPOR expression and thus lower sensitivity to PR-104A. Consistent with the hypoxic selectivity of PR-104A activation, PR-104A potency as quantified by the inverse of AUC for 10% survival (AUC_10_) was much higher (∼30-fold) under anoxic than aerobic conditions in SiHa and HCT116 (Figures 3D,E). This differential was greatly reduced when the AUC of PR-104H + M was used as the exposure variable, as shown for SiHa cells in Figure 3G. The slightly greater sensitivity of cells under anoxia was confirmed in SiHa cell suspensions exposed to synthetic PR-104H (Figures 4A,B), but for simplicity the average of the anoxic and oxic AUC_10_ values was used in the SR-PK/PD model.

#### The (pro)drug transport model

**Figure 5:**
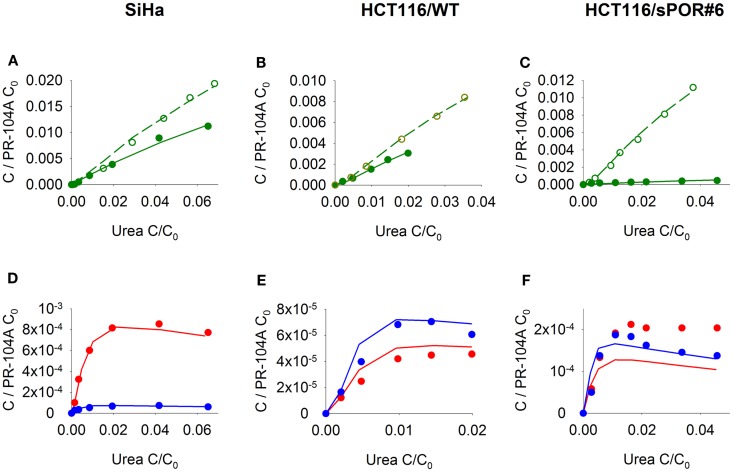
**Transport of PR-104A and reduced metabolites through MCLs grown from SiHa, HCT116/WT, or HCT116/sPOR#6 cells**. PR-104A was added to the donor side of MCLs (see Figure 1) under anoxia (closed symbols, solid lines) or oxia (open symbols, dashed lines). Graphs show receiver concentrations of PR-104A **(A–C)**, PR-104H [**(D–F)**; red] and PR-104M [**(D–F)**; blue], normalized to initial donor concentrations (*C*_0_) of PR-104A of 300 μM **(A,D)**, 650 μM **(B,E)**, and 250 μM **(C,F)**, and plotted against *C*/*C*_0_ of the ^14^C-urea internal standard to adjust for differences in MCL thickness. Symbols represent measured data for representative MCLs while lines show predictions of the (pro)drug transport model (Figure 2, Table S1 Supplementary Material) that best fitted the experiments overall (including cellular PK experiments illustrated in Figure 3).

**Figure 6:**
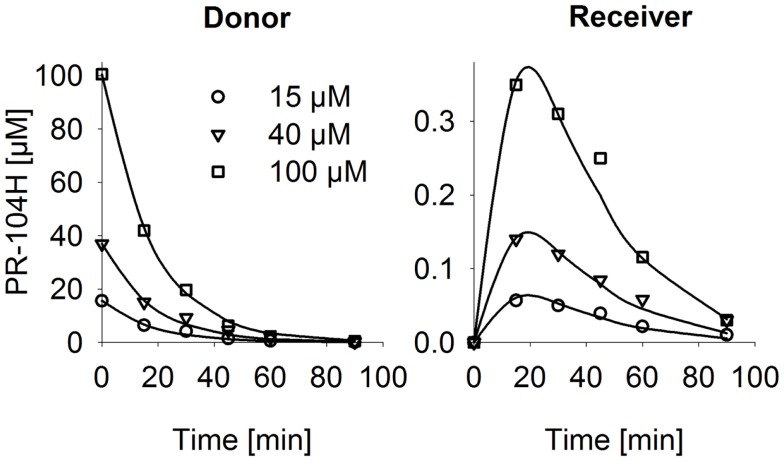
**PR-104H transport through SiHa MCLs**. PR-104H was added to the donor side of oxic SiHa MCLs that had been grown for 2 days only (to provide relatively thin MCLs; thickness 125 ± 3 μm). Graphs show measured (symbols) and model-estimated (lines) PR-104H concentrations on the donor side (left panel) and receiver side (right panel) of representative MCLs. Model fits used the parameters for PR-104H metabolism, instability, and membrane transfer estimates given in Table S1 Supplementary Material and a PR-104H diffusion coefficient of 1.06 ± 0.06 × 10^−6^ cm^2^/s.

Next, we investigated transport of PR-104A and its metabolites through MCLs following addition of PR-104A to the donor compartment. PR-104A penetration was suppressed by metabolic activation under anoxia (Figures 5A–C), which was accompanied by the appearance of PR-104H + M in the receiver compartment (Figures 5D–F), confirming that these metabolites can diffuse in a tissue-like environment after their intracellular generation from PR-104A. Notably, overexpression of sPOR in anoxic HCT116 MCLs increased concentrations of PR-104H + M in the receiver compartment ∼4-fold (Figure 5F), although markedly decreasing PR-104A penetration (Figure 5C). All data could be described using the two-compartment model described above, with the diffusion coefficients (*D_N_*) given in Table S1 in Supplementary Material. The PR-104H diffusion coefficient for SiHa was independently determined from measured MCL transport of PR-104H following its addition to the donor side (Figure 6).

The (pro)drug transport model was used to estimate the penetration half distances (*x*_1/2_) of active metabolites, by simulating their extracellular concentration-distance profiles at steady state when each compound is maintained at constant concentration on one side of an infinite planar slab. With this simplified geometry, *x*_1/2_ estimates were 128 μm (PR-104H) and 73 μm (PR-104M) in SiHa tissue and 33 μm (PR-104H) and 41 μm (PR-104M) in HCT116 tissue. This indicates that reduced metabolites may diffuse through several cell layers, once released from cells where they were produced.

### Estimation of the role of bystander effects using SR-PK/PD modeling

**Figure 7:**
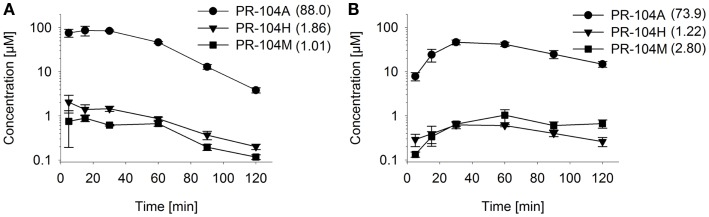
**Pharmacokinetics of PR-104A and its reduced metabolites in NIH-III nude mice**. Concentrations of PR-104A (●), PR-104H (▼), and PR-104M (■) in plasma **(A)** and HCT116 tumors **(B)** of NIH-III nude mice after i.p. administration of 562 μmol/kg of PR-104. Data represent mean ± SEM for three to four mice. Numbers in brackets are values for AUC_0–∞_in micromolar⋅hour as estimated by non-compartmental analysis using Phoenix WinNonlin (version 6.0; Pharsight, CA, USA).

**Figure 8:**
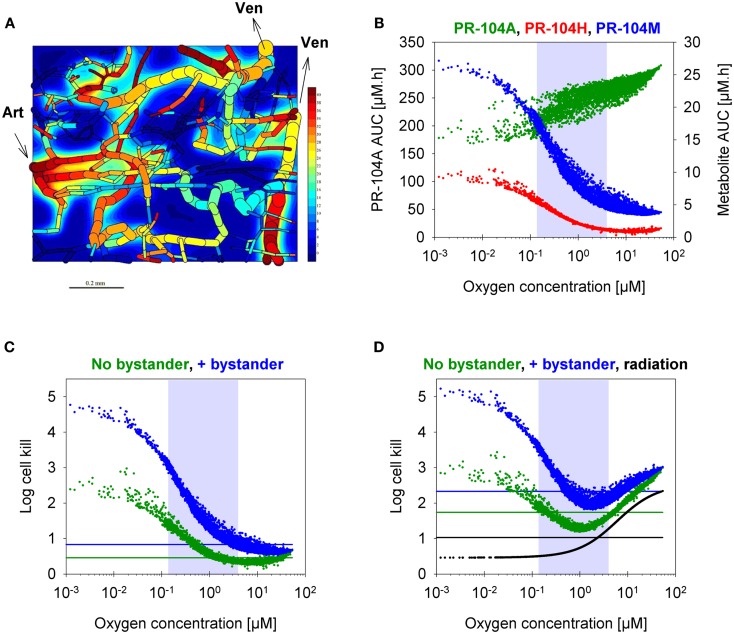
**SR-PK/PD model predictions for HCT116 tumors following i.p. administration of 562 μmol/kg PR-104**. **(A)** Contour plot of O_2_ (in mmHg) in a mid-plane section of the virtual 3D tumor microregion (990 × 810 × 150 μm), superimposed with the whole microvascular network projected onto the plane. Arrows indicate flow direction in the main feeding arteriole (Art) and the main draining venules (Ven). **(B–D)** PK/PD as a function of O_2_ in the tumor microregion: **(B)** intracellular AUC of PR-104A (left *y*-axis) and PR-104H and PR-104M (right *y*-axis), **(C)** Cell kill with and without a bystander effect, calculated using Eqs 4 and 5, respectively. The contribution of circulating metabolites was included to the “no-bystander” model by adding cell kill predicted for circulating PR-104H + M only (calculated by Eq. 4). **(D)** Cell kill by 10 Gy radiation (black) or by PR-104 in combination with radiation. Lines represent averaged cell kill for the whole tumor microregion. Gray shaded areas mark regions at intermediate O_2_ concentrations (between 0.13 and 4 μM, the $K_{{\text{O}}_{2}}$ – values for PR-104A and radiation, respectively).

**Figure 9:**
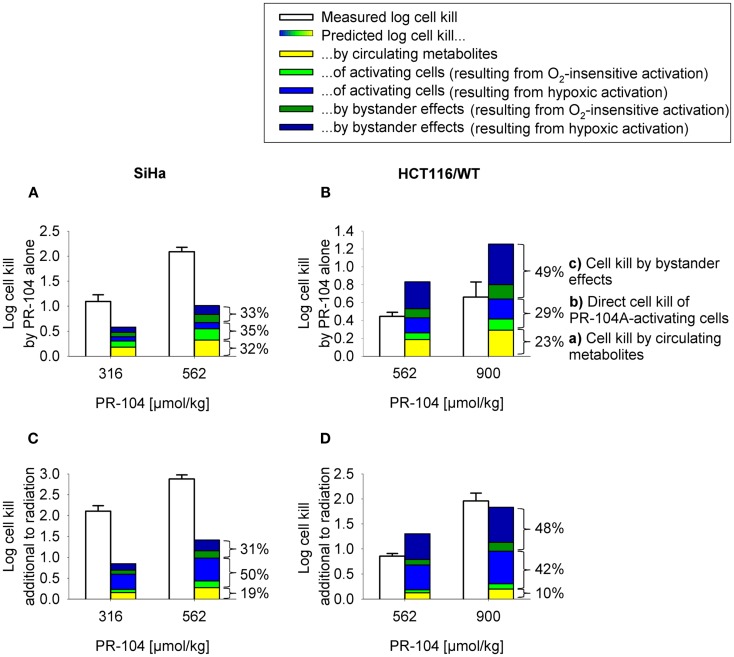
**Measured (white) and model predicted (colored) PR-104 antitumor activity**. **(A,B)** Monotherapy activity. **(C,D)** Activity in addition to radiation. Cell kill was measured in SiHa tumors grown in CD-1 nude mice [**(A,C)**; data from (20)] and HCT116 tumors grown in NIH-III nude mice (**B,D)**, 18 h after i.p. administration of PR-104 with or without whole-body irradiation 5 min before PR-104 dosing [**(C)**: 15 Gy; **(D)**: 10 Gy]. Data show mean ± SEM for four or five mice. Predicted values represent average log cell kill in the tumor microregion. Contributions of mechanisms **(a–c)** were dissected by: **(a)** setting plasma PR-104A AUC to 0, **(b)** relating cell kill to prodrug AUC (Eq. 5), and **(c)** subtracting **(a,b)** from total predicted cell kill. Cell kill resulting from hypoxic (blue) or O_2_-insensitive (green) PR-104A activation was distinguished by setting *k*_met_*_A_*_,min_ to 0.

To assess the impact of metabolite diffusion on cell killing in tumors, the above PK/PD parameters were used to calculate prodrug/metabolite exposure and resulting cell killing at each point of a digitized FaDu tumor microregion. By adjusting inflow pO_2_, the hypoxic fraction (at <1 μM O_2_) in this region was matched to the pimonidazole-positive fraction measured in HCT116 tumors (23.0%) or SiHa tumors (12.3%) in our lab (22). The frequency distributions of O_2_ concentrations in the tumor microregion are shown in Figure S1C,D in Supplementary Material. The spatial O_2_ distribution in the microregion used to model HCT116 tumors is shown in Figure 8A. Measured PR-104 plasma PK was used to define unbound AUC of PR-104A/H/M in all inflowing vessels of the tumor microregion. Notably, the PK was different in the mouse strains used to grow SiHa tumors [CD-1 nude mice (33)] and HCT116 tumors [NIH-III nude mice; Figure 7], with NIH-III nude mice showing ∼fivefold lower levels of circulating metabolites at equivalent dose. Therefore low-AKR1C3 HCT116 tumors in NIH-III nude mice represent a good model to evaluate the impact of bystander effects resulting from PR-104A activation in hypoxic tumor regions. SR-PK/PD simulations for this tumor model showed decreasing PR-104A and increasing PR-104H + M exposure (AUC) with decreasing O_2_ concentrations (Figure 8B). The contribution of bystander effects was distinguished by comparison of the “no-bystander” and “+bystander” simulations that relate cell killing to prodrug and metabolite AUC, respectively (see Eqs 4 and 5). The “+bystander” model predicted higher killing across the entire tumor microregion (Figure 8C), and improved complementarity with radiation (Figure 8D). Bystander effects were estimated to contribute ∼50% of predicted overall cell killing in HCT116 tumors grown in NIH-III nude mice and ∼30% of activity in SiHa tumors grown in CD-1 nude mice, with very similar estimates for PR-104 monotherapy activity and killing additional to radiation (Figure 9). Remaining activity was due to direct killing of prodrug-activating cells and killing of perivascular cells by circulating metabolites with the contribution of the latter higher in CD-1 nude mice because of the higher PR-104H + M plasma AUC in this strain. The relative contribution of overall (direct + bystander) killing resulting from O_2_-sensitive one-electron reduction (30% in SiHa and 55% in HCT116 tumors) and overall killing arising from O_2_-insensitive AKR1C3-mediated two-electron reduction (38% in SiHa and 22% in HCT116 tumors) was influenced by hypoxic fraction (higher in HCT116 tumors) and AKR1C3 expression (higher in SiHa tumors).

### Evaluation of the SR-PK/PD model by comparison of measured and model-estimated PR-104 antitumor activity

**Figure 10:**
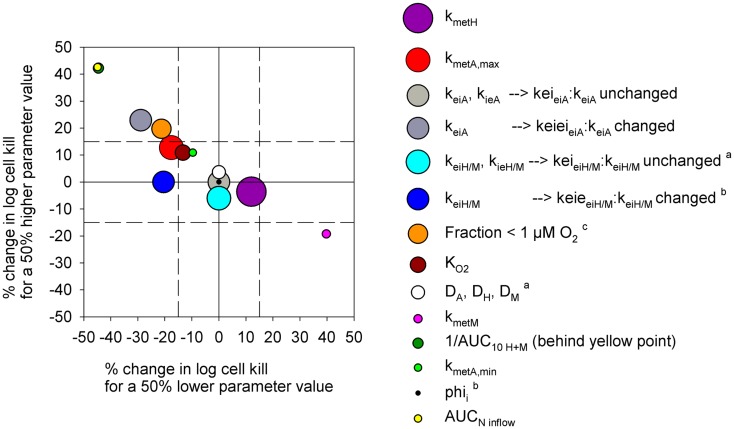
**Sensitivity analysis of the PR-104 SR-PK/PD model for HCT116 tumors**. Percentage of change in model-estimated monotherapy activity (at 562 μmol/kg PR-104) resulting from a 50% increase in parameter values relative to the default model values of Table S1 in Supplementary Material (*y*-axis) plotted versus the respective % change in killing resulting from a 50% decrease in parameter values (*x*-axis). Symbols represent individual parameters, with the symbol area proportional to the CV of the estimated parameter values (highest CV 59.6% for *k*_met_*_H_*). No CV estimate was available for *k*_met_*_A_*_,min_ and *AUC_N_*_inflow_ (with *N* denoting A, H, or M). ^a^No predictions were available for a 50% lower parameter value. ^b^No predictions were available for a 50% higher parameter value. ^c^ The fraction at <1 μM O_2_ was modulated by changing inflow pO_2_.

In order to test the predictive ability of the SR-PK/PD model, we utilized previous data for clonogenic cell killing in SiHa tumors (20) and also evaluated activity in HCT116 tumors, 18 h after treatment with two dose levels of PR-104, either alone or immediately after a single radiation dose. Higher PR-104 doses were tested with HCT116 which was the less sensitive of the two tumor models (Figure 9). The SR-PK/PD model predictions for overall (averaged) cell kill were broadly similar to measured values, although the model under-estimated killing in SiHa tumors while over-estimating killing in HCT116 tumors. This might reflect missing information about the biology of these tumors, or errors in the model parameters. To evaluate the sensitivity of the HCT116 SR-PK/PD model to parameter errors, we varied each parameter ±50%; this did not change predictions for PR-104 monotherapy activity by more than 45% in any instance (Figure 10), demonstrating that the model is reasonably robust. Predictions could be matched to experimental results by a 50% decrease in the rate constant for PR-104A uptake, *k*_*ei*_*_A_*, which had a relatively high CV of 32%, or by a 50% decrease in reduced metabolite potency (1/AUC_10_*_H_*_+_*_M_*; CV 7.3%) and inflow AUC of PR-104A/H/M (error estimate not available). Model predictions were also not highly sensitive to the specific features of the FaDu network; similar cell killing was predicted when we used a mapped network from a rat R3230Ac tumor (28) (with O_2_ inflow adjusted to achieve similar oxygenation in the FaDu and R3230Ac networks) even though the latter network showed 1.8-fold higher total blood inflow per tissue volume and a 1.5-fold higher median distance to nearest vessel (Figure S1 in Supplementary Material).

**Figure 11:**
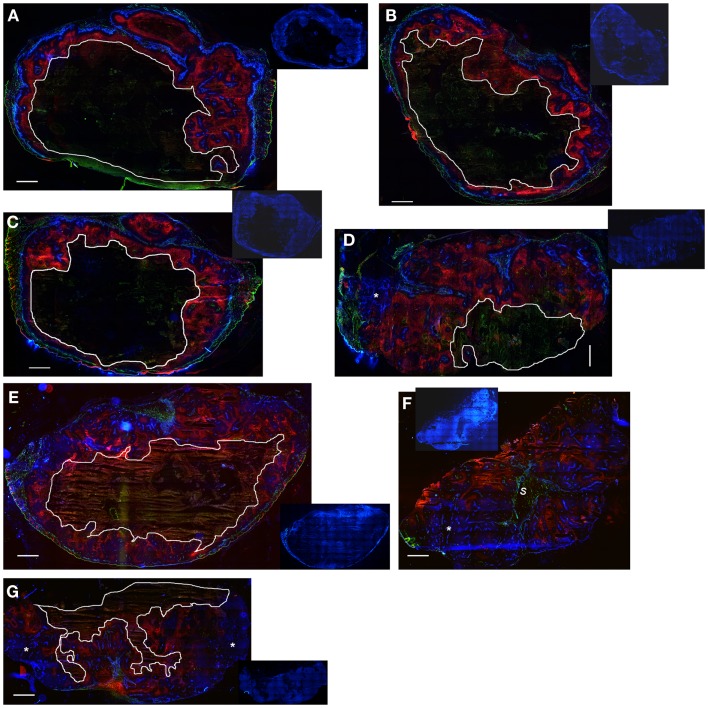
**Hypoxia, vasculature, and perfusion in SiHa and HCT116 tumors**. Photomicrographs show overlaid images of the perfusion marker Hoechst 33342 (blue), the hypoxia marker EF5 (red), and the vascular marker CD31 (green) on frozen sections of SiHa tumors **(A–D)** and HCT116 tumors **(E–G)**. Inset pictures show DAPI nuclear staining on the same **(E–G)** or adjacent sections **(A–D)**. Areas of necrosis are encircled by white lines. s, stromal connective tissue; *large well-perfused regions. Scale bars represent 1 mm.

Notably, the model over-estimated PR-104 monotherapy activity in HCT116 tumors, while providing better predictions of PR-104-mediated killing additional to radiation with either networks (as shown based on the FaDu network in Figures 9B,D). This might be explained by the presence of large well-oxygenated regions in HCT116 tumors, which are efficiently killed by radiation but not by treatment with PR-104 alone because they are beyond the reach of local bystander effects. Imaging of the hypoxia marker EF5 and the perfusion marker Hoechst 33342 on frozen tumor sections revealed that HCT116 tumors do indeed contain large well-perfused regions without EF5-staining (>1 mm^2^; Figure 11). In SiHa tumors, such large well-oxygenated regions were less common (Figure 11).

### Modulation of hypoxia and reductase activity to further evaluate the SR-PK/PD model

**Figure 12:**
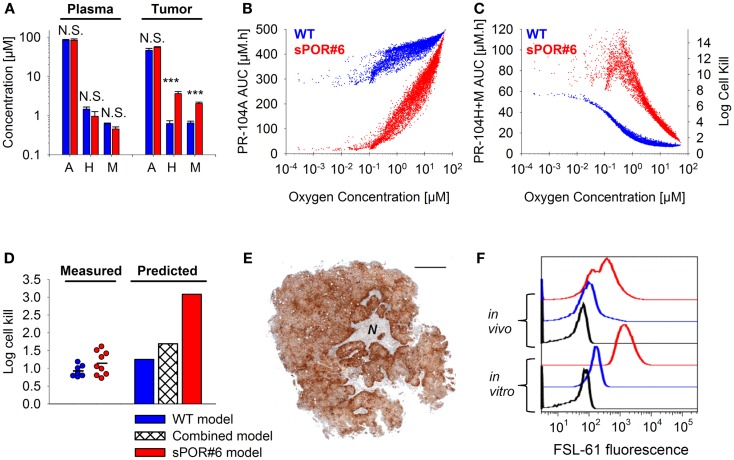
**Effect of POR overexpression on PR-104 PK/PD**. **(A)** Concentrations of PR-104A, PR-104H, and PR-104M in tumor and plasma 30 min after i.p. administration of 562 μmol/kg PR-104 to NIH-III nude mice with HCT116/WT tumors (blue) or HCT116/sPOR#6 tumors (red). Data represent mean ± SEM for four to five mice. N.S. not significant; ***P < 0.001, *t*-test. **(B,C)** SR-PK/PD model predictions for HCT116/WT and HCT116/sPOR#6 at a PR-104 dose of 900 μmol/kg. Graphs show the distribution of intracellular PR-104A AUC **(B)**, intracellular PR-104H + M AUC and resulting cell kill **(C)** as a function of O_2_ in the tumor microregion. **(D)** Predicted average log cell kill for HCT116/WT, HCT116/sPOR#6, and a combined model with a 1:2 ratio of WT and sPOR#6 in separate regions (see text). Symbols represent measured killing in individual tumors 18 h after i.p. administration of 900 μmol/kg PR-104, with a line marking the mean. **(E)** Histological section of a representative HCT116/sPOR#6 tumor showing POR immunostaining. Areas of necrosis are marked with *N*. Scale bar is 1 mm. **(F)** Representative flow cytometry histograms of cells from monolayer culture (*in vitro*) or from tumor xenografts (*in vivo*) after incubation with 300 μM FSL-61 for 3 h under anoxic conditions (blue: WT, red: sPOR#6) or as a control under aerobic conditions (black, WT).

The SR-PK/PD model was tested further by modulating two parameters to which the model is moderately sensitive (Figure 10). Firstly, tumors grown from HCT116/sPOR#6 cells were used to evaluate the effect of increasing the rate constant for anoxic PR-104A reduction *k*_met_*_A_*_,max_. Average tumor concentrations of PR-104H + M 30 min after i.p. administration of 562 μmol/kg PR-104 were significantly higher in HCT116/sPOR#6 than WT tumors (Figure 12A), confirming functional expression of sPOR *in vivo*. This did not increase plasma concentrations of PR-104H + M, consistent with earlier studies demonstrating that systemic exposure to these metabolites is primarily due to hepatic (not intra-tumor) activation of PR-104A (33). Therefore SR-PK/PD in HCT116/sPOR#6 tumors was simulated with unchanged drug inflow AUCs, using a 20.4-fold increased *k*_met_*_A_*_,max_ as determined in HCT116/sPOR#6 cell suspensions. In the model, the increased metabolic consumption substantially impaired PR-104A penetration into hypoxic regions (Figure 12B), but increased levels of reduced metabolites throughout the tumor microregion (Figure 12C), causing ∼2.5-fold higher average predicted cell kill relative to WT (Figure 12D). In contrast, measured PR-104 monotherapy activity at a dose of 900 μmol/kg was not significantly different between HCT116/WT and HCT116/sPOR#6 tumors (*P* = 0.187, *t*-test; Figure 12D). A likely explanation is the heterogeneity of sPOR expression in HCT116/sPOR#6 tumors as demonstrated by immunohistochemistry, with low-sPOR cells often found at a distance of 100–300 μm from high-sPOR cells (Figure 12E) and thus expected to be beyond the range of bystander metabolites. Further evidence for heterogeneity of sPOR expression *in vivo* includes the lack of puromycin resistance in approximately one third of cells recovered from HCT116/sPOR tumors (SF in puromycin medium 67.2 ± 2.4%, *n* = 3), suggesting loss of expression of the bicistronic *sPOR*-IRES-*pac* mRNA encoding *sPOR* and the puromycin resistance gene *pac* (puromycin *N*-acetyltransferase). In addition, anoxic one-electron reduction of the fluorogenic probe FSL-61 [which correlates with anoxic PR-104A reduction in tumor cell lines (43)] demonstrated marked heterogeneity in cells from HCT116/sPOR#6 tumors with ca. one third having one-electron-reductase activity similar to WT cells and remaining cells showing FSL-61 fluorescence intermediate between WT and sPOR#6 cells *in vitro* (Figure 12F) As a simple way of modeling the macroregional heterogeneity, we assumed that HCT116/sPOR#6 tumors comprise two separate, non-communicating compartments with *k*_met_*_A_*_,max_ equivalent to sPOR#6 in culture (possibly still over-estimating one-electron reduction of PR-104A) and at the WT level in a 2:1 ratio. This combined model provided much better prediction of PR-104 activity in HCT116/sPOR#6 tumors (Figure 12D).

**Figure 13:**
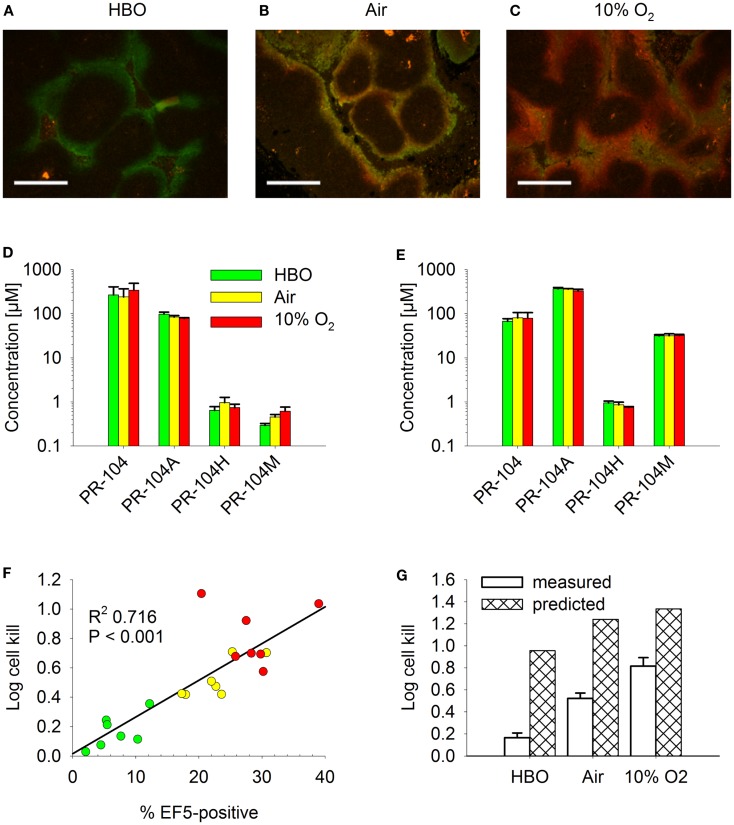
**Effect of O_2_ modulation on cell killing in HCT116/sPOR#6 tumors**. **(A–C)** Overlay of micrographs of the hypoxia markers EF5 [red; reporting hypoxia during treatment with **(A)** hyperbaric oxygen (HBO), **(B)** air, or **(C)** 10% O_2_] and pimonidazole (green; reporting pre-existing hypoxia) from representative sections of HCT116/WT tumors. Tumor-bearing NIH-III nude mice were dosed i.p. with 60 mg/kg pimonidazole 2 h prior to i.p. administration of 60 mg/kg EF5 followed by treatment with respiratory gasses for 1.5 h. Scale bars are 300 μm. **(D–G)** PR-104 PK and PD as assessed after co-administration of 562 μmol/kg PR-104 and 30 mg/kg EF5 i.p. to tumor-bearing NIH-III nude mice, followed by treatment with HBO (green), air (yellow), or 10% O_2_ (red) for 30 min **(D,E)** or 2 h **(F,G)**. **(D,E)** Concentrations of PR-104 and metabolites in plasma **(D)** and liver **(E)**. Data represent mean ± SEM for five (air + 10% O_2_) or six mice (HBO). **(F)** Log cell kill in individual tumors as a function of EF5-positive fraction, with linear regression line. **(G)** mean ± SEM of the data shown in F (measured) and estimates calculated by the combined model for HCT116/sPOR#6 tumors (predicted; see Figure 12 legend), with oxygenation in the virtual tumor microregion for each condition adjusted to achieve a fraction of cells <1 μM O_2_ corresponding to the mean EF5-positive tumor fraction measured in this experiment (see Figure S2 in Supplementary Material for frequency and distribution of O_2_ concentrations).

For a further evaluation of the SR-PK/PD model, tumor hypoxia was modulated by exposing tumor-bearing mice to hyperbaric oxygen, air or 10% O_2_ following administration of PR-104 and/or EF5. The respiratory gasses modulated hypoxia in HCT116/WT tumors as demonstrated by dual imaging with the hypoxia markers pimonidazole and EF5 (Figures 13A–C), but did not significantly change concentrations of PR-104, PR-104A, and reduced metabolites in plasma (Figure 13D) or liver (Figure 13E) 30 min after i.p. administration of PR-104. This is consistent with previous data indicating that hepatic metabolism of PR-104A in mice is independent of hypoxia (33), and suggests that tumor input of PR-104A and metabolites is unaffected by the respiratory gases. PR-104 monotherapy activity in HCT116/sPOR#6 tumors correlated with the EF5-positive fraction (Figure 13F). The combined model introduced above (one third of HCT116/sPOR#6 tumor regions with PR-104A activation similar to WT) predicted this trend, although over-estimating killing (Figure 13G). Independent of the model, the observation that killing was greater than accounted for by the proportion of EF5-positive cells (e.g., 1 log cell kill, i.e., 90% killed fraction at an EF5-positive fraction of ∼40%; Figure 13F) can be considered further evidence for bystander effects.

## Discussion

Our data clearly demonstrate that the active metabolites of PR-104A can efflux from cells (Figures 3A–C), as previously reported (13), and are able to diffuse through tissue-like MCLs following their addition to extracellular medium (Figure 6) and following their production from PR-104A in anoxic MCLs (Figures 5D–F). The experimental observations alone, however, do not indicate the extent to which these diffusible metabolites contribute to antitumor activity. Consequently, we developed a SR-PK/PD model that calculates the spatial distribution of PR-104A, its metabolites and their pharmacodynamic effect in a realistic tumor microregion. The model considers intra- and extracellular compartments to account for cell membranes acting as a barrier to the diffusion of active metabolites after their intracellular formation from PR-104A. Using this model, we have shown that bystander effects play a major role in PR-104 activity in tumor xenografts (Figure 9). The contribution of bystander effects was distinguished by comparison of the “no-bystander” and “+bystander” simulations that relate cell killing to prodrug and active metabolite AUC, respectively (Eqs 4 and 5), according to the PK/PD relationships determined in single cell suspensions. The “no-bystander” model predicted less killing across the entire tumor microregion, even in the most hypoxic regions (Figure 8C), which may seem surprising given that cell kill in anoxic single cell suspensions could be predicted equally well using the PR-104A or metabolite PK/PD relationships (Figures 3E,H). This difference is a consequence of the failure of the “no-bystander” model to account for the cell density dependence of killing that follows from the ability of active metabolites to diffuse out of PR-104A-activating cells, leading to a local rise of metabolite concentrations in tumor tissue relative to single cell suspensions. These short-scale bystander effects (diffusion across the plasma membrane and uptake by adjacent cells) and medium-scale bystander effects (diffusion to better-oxygenated regions; in the current model, paracellular only) improved complementary killing by PR-104 and radiation by partially compensating for inefficient PR-104A activation at O_2_ concentrations low enough to cause radioresistance (∼0.1–4 μM O_2_; Figure 5D). There are potentially also larger-scale blood-borne bystander effects resulting from diffusion of active metabolites into blood vessels and killing of perivascular cells in downstream tumor regions.

Overall clonogenic killing in tumors estimated by the SR-PK/PD model was in broad agreement with measured values, although the model under-predicted activity in SiHa tumors and over-predicted killing in HCT116 tumors (Figure 9). The fact that the model was biased in opposite directions for HCT116 and SiHa tumors suggests that the discrepancies are due to biological factors that differ between the two tumor types, and that are not currently captured by the model. One possible missing element is the apparent O_2_-dependence of reduced metabolite potency in SiHa cells (Figure 3G), which warrants further investigation. Other factors could include differences between model parameters *in vivo* relative to those determined *in vitro*. Parameters in question are $K_{{\text{O}}_{2}}$ (given theoretical arguments that $K_{{\text{O}}_{2}}$ may be cell density dependent (9), although this has yet to be tested experimentally) and the parameters for PR-104A metabolism and cellular sensitivity to active metabolites (e.g., due to potential differences in expression of PR-104A reductases and DNA damage response pathways in tumors versus *in vitro*). However, preliminary experiments showed that PR-104A metabolism and cytotoxicity is similar in cells from *in vitro* culture and from tumor xenografts despite slight apparent differences in AKR1C3 protein expression (Figure S3 in Supplementary Material). An additional factor we have considered is possible intra-tumor generation of PR-104A from PR-104 or from its *O*-β-glucuronide PR-104G (which is a minor metabolite in mice but the major PR-104 metabolite in humans (48). Studies with oxic SiHa MCLs demonstrate that PR-104A is generated from PR-104, but not from PR-104G (Figure S4 in Supplementary Material). Thus intra-tumor PR-104 hydrolysis could potentially increase PR-104A exposure in the tumor although this is unlikely to fully account for the ∼twofold underprediction of PR-104 activity in SiHa tumors, given that the PR-104 AUC constitutes only ∼30% of the PR-104A AUC in plasma of CD-1 nude mice dosed i.p. with PR-104 (15).

Finally, the FaDu tumor microregion used in the SR-PK/PD model has a specific microvascular geometry and blood flow distribution that may not represent that of SiHa and HCT116 tumor xenografts. However, in the only other digitized tumor microvascular network with measured blood flows, based on a mapped region of a rat R3230Ac tumor (28), log cell kill predictions were within 24% of those for the FaDu network (Figure S1 in Supplementary Material). A greater limitation of both microregions may be their small volume (0.12 mm^3^ for FaDu and 0.066 mm^3^ for R3230Ac) that does not adequately account for macroregional heterogeneity in tumor oxygenation. Importantly, such heterogeneity was observed in HCT116 tumors, showing large well-perfused areas without EF5-staining (>1 mm^2^; Figure 11), in which cells are expected to be killed by radiation but not by treatment with PR-104 alone. This might account for the better prediction of PR-104 activity when combined with radiation (which will be effective against these extended oxic regions) in HCT116 tumors (Figures 9B,D). Larger-scale networks with mapped spatial distributions of hypoxia would be required to improve SR-PK/PD modeling in the future. In addition, a complete description would need to consider the implications of cycling hypoxia (49), which is not currently incorporated in the steady-state SR-PK/PD model.

The results of this study have several implications for the clinical use of PR-104. Firstly, the finding that O_2_-sensitive and O_2_-insensitive PR-104A activation pathways make comparable contributions to PR-104 monotherapy activity (Figure 9) argues for the use of biomarkers of both pathways (expression of AKR1C3, and one-electron-reductases in hypoxic cells) in the context of PR-104 therapy. Secondly, the correlation between PR-104 monotherapy activity and EF5-positive fraction in HCT116/sPOR#6 tumors (Figure 13F) confirms the dependence of PR-104 activity on hypoxia-selective PR-104A activation and suggests that EF5 or other 2-nitroimidazole hypoxia probes could potentially be used as predictive biomarkers for PR-104. In this context, the 2-nitroimidazole probes may be acting as reporters of one-electron-reductase activity as well as hypoxia, as demonstrated for the tirapazamine analog SN30000 (42). Thirdly, MCL data (Figures 5C,F) and SR-PK/PD predictions (Figures 12B–D) for HCT116/sPOR#6 suggest that PR-104 can be applied in human tumors with high expression of one-electron-reductases without the resulting limitation on PR-104A penetration compromising therapeutic activity. Fourthly, the lack of a significant difference in PR-104 monotherapy activity between HCT116/WT tumors and HCT116/sPOR#6 tumors (with non-uniform sPOR expression; Figure 12), implies that high one-electron-reductase activity will not increase PR-104 antitumor activity if there is macroregional heterogeneity in expression over spatial scales exceeding those of bystander effects. A full treatment of this problem would require mapping of spatial heterogeneity of reductases, which is likely to be an important issue in human tumors given the heterogeneity of POR expression in histological sections of individual human tumors (19). Finally, macroregional variations in hypoxia are likely to limit the reach of bystander effects, implying that local diffusion of active metabolites will not eliminate the need to combine PR-104 (or other HAP) with agents that provide complementary killing of aerobic cells.

To our knowledge this is the first study to model bystander killing in tumor tissue based on measured parameters for metabolism, diffusion and cytotoxicity of prodrug metabolites. Unlike a purely experimental approach, SR-PK/PD modeling has the potential to dissect the parameters underlying tissue penetration of a prodrug and its metabolites, thus providing an opportunity to identify features that could be optimized by drug design.

## Authors contribution

Annika Foehrenbacher, Kashyap Patel, William R. Wilson, and Kevin O. Hicks conceived and designed the experiments. Kashyap Patel and Annika Foehrenbacher performed experiments with SiHa and HCT116 cell lines, respectively. Maria R. Abbattista and Chris P. Guise generated and characterized the cell line HCT116/sPOR#6. Timothy W. Secomb developed the Green’s function method and wrote the program to simulate multiple intracellular and extracellular solutes. Kevin O. Hicks and Annika Foehrenbacher determined the model parameters by fitting the model equations to experimental data. Annika Foehrenbacher designed and ran the simulations and Annika Foehrenbacher, Kevin O. Hicks, and William R. Wilson analyzed the data. Annika Foehrenbacher assembled the figures, table, and manuscript. Annika Foehrenbacher, Kevin O. Hicks, William R. Wilson, Timothy W. Secomb, and Kashyap Patel wrote the paper.

## Conflict of Interest Statement

William R. Wilson is an inventor on patents relating to PR-104. The other co-authors declare that the research was conducted in the absence of any commercial or financial relationships that could be construed as a potential conflict of interest.

## Supplementary Material

The Supplementary Material for this article can be found online at: http://www.frontiersin.org/Pharmacology_of_Anti-Cancer_Drugs/10.3389/fonc.2013.00263/abstract
