# Supplementary material for: The Role of Bystander Effects in the Antitumor Activity of the Hypoxia-Activated Prodrug PR-104
Source: Front Oncol. 2013 Oct 7;3:263. doi: 10.3389/fonc.2013.00263 (PMC3791487; doi:10.3389/fonc.2013.00263)
Supplement: Supplementary file 1 [file 65618_Wilson_Presentation1.PDF]

Supplementary Material

Derivation of the equations describing the PK of PR-104A and metabolites

Concentrations of PR-104A, PR-104H and PR-104M in single cell suspension, MCL and the tumor microregion were described by an intracellular/extracellular 2-compartment (pro)drug transport model (Figure 2). The equations of the model were derived as follows.

All transport equations are expressed in terms of amounts per total volume (intracellular + extracellular). φ_i and φ_e are the intra- and extracellular volume fractions, respectively, with $\varphi_e = 1-\varphi_i$ and $N = A$, H or M denoting the three solutes.

The flux q_N across a cell membrane with the membrane permeability P_N (units of velocity) is given (in units of amount per volume per time) by:

$$q_N = P_N A(C_{eN} - C_{iN}) \tag{S1}$$

where C_{eN} and C_{iN} are concentrations in extracellular and intracellular compartments and A is the cell area per total volume (units of 1/length) with:

$$A = A_c\varphi_i. \tag{S2}$$

where A_c is the surface-to-volume ratio of the cells. The rate constant for transfer between the extracellular and intracellular compartments k_N is defined by:

$$k_N = P_N A_c \tag{S3}$$

Substitution of equations S2 and S3 into equation S1 gives:

$$q_N = \varphi_i k_N(C_{eN} - C_{iN}) \tag{S4}$$

Unequal partitioning of concentrations between compartments can be allowed by introducing two different rate constants for transfer from the extracellular to the intracellular compartment (k_{eiN}) and the reverse transfer (k_{ieN}):

$$q_N = \varphi_i(k_{eiN}C_{eN} - k_{ieN}C_{iN}) \tag{S5}$$

The changes in extracellular and intracellular concentrations resulting from partitioning are thus given by:

$$\varphi_e\frac{\partial C_{eN}}{\partial t} = -q_N \tag{S6}$$

and

$$\varphi_i\frac{\partial C_{iN}}{\partial t} = q_N. \tag{S7}$$

The diffusive flux $\boldsymbol{j}_N$ in the extracellular compartment (in units of amount per area per time) is

$$\boldsymbol{j}_N = -D_N\nabla C_{eN} \tag{S8}$$

where D_N represents the diffusion coefficient. In the absence of solute production or removal, conservation of mass gives:

$$\varphi_e\frac{\partial C_{eN}}{\partial t} + \nabla\cdot\boldsymbol{j}_N = 0 \Rightarrow \varphi_e\frac{\partial C_{eN}}{\partial t} = D_N\nabla^2 C_{eN} \tag{S9}$$

With a rate constant for instability in both compartments $k_{instabN}$ and a rate constant for metabolism in the intracellular compartment k_{metN}, the overall equations for extracellular and intracellular concentrations are:

$$\varphi_e \frac{\partial C_{eN}}{\partial t} = D_N \nabla^2 C_{eN} - \varphi_i (k_{eiN} C_{eN} - k_{ieN} C_{iN}) - \varphi_e k_{instabN} C_{eN} \qquad \text{(S10)}$$

$$\varphi_i \frac{\partial C_{iN}}{\partial t} = \varphi_i (k_{eiN} C_{eN} - k_{ieN} C_{iN}) - \varphi_i k_{metN} C_{iN} - \varphi_i k_{instabN} C_{iN} + \varphi_i r_N \qquad \text{(S11)}$$

where r_N is the rate of metabolic production with $r_A = 0$, $r_H = \varphi_i k_{metA} C_{iA}$ and $r_M = \varphi_i k_{metH} C_{iH}$.

Table S1. Parameters and variables of the PR-104 SR-PK/PD model.
Parameters for O_2 transport and radiosensitivity were as previously reported except O_2 inflow was adjusted to achieve the required hypoxic fraction in the microregion. Parameters for transport of PR-104A, PR-104H and PR-104M were determined by fitting one-dimensional time-dependent solution of equations S10 and S11 to cellular PK and MCL data. The potency of PR-104A and its reduced metabolites was determined by linear regression of cellular PK/PD relationships.

Parameter	Unit	Estimate for		Meaning, references & comments
		SiHa	HCT116	
**O_2 transport parameters**				
Q	nl/min	40	40	Total blood flow to microregion = sum of blood flow in all feeding arterioles of the tumor microvascular network. Flow in remaining vessels is scaled from measured values by the factor Q/(total measured inflow).
pO_2[a]	mm Hg	50/79	40/54	O_2 partial pressure in inflowing vessels of the FaDu/R3230Ac microvascular network. These values were adjusted to achieve a certain extent of hypoxia.
P_{50}	mm Hg	38	38	O_2 partial pressure for half saturation of hemoglobin (1).
n		3.0	3.0	Hill coefficient (1).
C_0	cm^3 O_2/mm Hg	0.5	0.5	O_2 binding capacity of red blood cells (2).
H_D		0.4	0.4	Discharge hematocrit (3).
α	cm^3 O_2/cm^3/ mm Hg	3.1×10^{-5}	3.1×10^{-5}	O_2 solubility (3).
αD_{O2}	cm^3 O_2/cm/s/ mm Hg	4.2×10^{-10}	4.2×10^{-10}	Krogh diffusion coefficient for O_2 diffusion (3).
M_0	cm^3 $O_2/cm^3/s$	5×10^{-4}	5×10^{-4}	Rate of O_2 consumption when supply is not limiting. The range of M_0 values in R3230Ac tumors was 1.4 - 3.7 x 10^{-4} (4). Here, M_0 was set to a slightly higher value to achieve a sufficient hypoxic fraction.
K_{mO2}	mm Hg	1.0	1.0	Michaelis constant for O_2 consumption (5).

**Radiosensitivity**				
α_H	Gy^{-1}	0.0663	0.0479	Proportionality constants for the linear-quadratic model under hypoxia. Values were estimated to provide overall radiation-induced cell kill in the tumor microregion in the range measured for SiHa tumors (6) and HCT116 tumors (1.04 ± 0.11, n = 4). The corresponding α:β ratios of 8.5 Gy (SiHa) and 3 Gy (HCT116) are within the range of α:β ratios (1.2 - 87 Gy) determined for experimental tumors (7).
β_H	Gy^{-2}	0.0028	0.00574	
$OER_{\alpha,max}$ = $OER_{\beta,max}$		2.8	2.8	Maximal O_2 enhancement ratios for the α and β components of the linear-quadratic model (8).
K_{ms}	µM	4.2	4.2	O_2 concentration for half maximum radiosensitivity calculated from (8).
D_r	Gy	15	10	Radiation dose used in tumor xenograft experiments.
**Inflow of PR-104A, PR-104H + PR-104M**[a]				
$AUC_{A\ inflow}$	µM.h	45.9	61.5	Inflow AUC = unbound AUC estimated from plasma PK measured after i.p. administration of 562 µmol/kg PR-104 to CD-1 nude mice (9) and NIH-III nude mice (Figure 7), the host mice used for SiHa tumors and HCT116 tumors, respectively. Unbound AUC was calculated from $AUC_{0-\infty}$ estimates using the unbound fraction of 0.699 measured for PR-104A in murine plasma (Patel et al., 2011), assuming equivalent protein binding for PR-104H and PR-104M. Dose-linearity of AUCs was assumed for simulations with doses other than 562 µmol/kg. Since circulating PR-104H+M in CD-1 nude mice were only measured after dosing of PR-104 i.v. (14), AUC values of these metabolites were estimated assuming equivalent AUC ratios of PR-104H/PR-104A = 0.036 and PR-104M/PR-104A = 0.153 after i.p. dosing.
$AUC_{H\ inflow}$	µM.h	1.63	1.30	
$AUC_{M\ inflow}$	µM.h	7.01	0.71	

**Cellular PK model parameters**[b]				
$k_{instabA}$	s^{-1}	1.83×10^{-5}	1.83×10^{-5}	Non-enzymatic loss of PR-104A (6), PR-104H and PR-104M (data not shown), measured after their addition to culture medium without cells at 37 ºC. All k_{instab} were independent of oxygen and drug concentration.
$k_{instabH}$	s^{-1}	9.33×10^{-4}	9.33×10^{-4}	
$k_{instabM}$	s^{-1}	1.67×10^{-3}	1.67×10^{-3}	
K_{O2}	μM	0.126 ± 17%	0.126 ± 17%	O_2 concentration for half-maximum PR-104A activation, assumed to be equal to the O_2 concentration for half-maximum PR-104A cytotoxicity determined in SiHa single cell suspensions (6), based on the dependence of cytotoxicity on PR-104A activation observed *in vitro* at pharmacologically relevant PR-104A concentrations (Singleton et al., 2009)
$k_{metA,max}$	s^{-1}	0.0159 ± 36%	9.00×10^{-4} ± 40%	Rate constants for metabolism of PR-104A to PR-104H under anoxia (max) and aerobic conditions (min). All values were fitted to single cell suspension data apart from the $k_{metA,min}$ for SiHa, which was assumed to be 0.061 x $k_{metA,max}$ because 0.061 was the value reported for the O_2-insensitive fraction of PR-104A cytotoxicity in SiHa cell suspensions (6).
$k_{metA,min}$	s^{-1}	9.70×10^{-4}	2.07×10^{-5}	
k_{metH}	s^{-1}	5.17×10^{-3} ± 23%	1.5×10^{-2} ± 60%	Rate constant for metabolism of PR-104H to PR-104M.
k_{metM}	s^{-1}	0.0120 ± 37%	3.0×10^{-3} ± 5.1%	Rate constant for metabolism of PR-104M to downstream products.
k_{eiA}	s^{-1}	0.0348 ± 32%	5.0×10^{-2} ± 32%	Rate constants for transfer from the extracellular to the intracellular compartment (k_{eiN}) and vice versa (k_{ieN}) for PR-104A, PR-104H and PR-104M. Values were assumed to be equal for PR-104H and PR-104M because of their similar physicochemical properties. The k_{eiN}:k_{ieN} ratio was assumed to be 1 for SiHa and >1 for HCT116, based on observed ratios of intra- to extracellular concentrations in single cell suspensions at steady-state.
k_{eiH}	s^{-1}	0.0160 ± 20%	1.5×10^{-2} ± 31%	
k_{eiM}	s^{-1}	0.0160 ± 20%	1.5×10^{-2} ± 33%	
k_{ieA}	s^{-1}	0.0348 ± 32%	1.0×10^{-2} ± 23%	
k_{ieH}	s^{-1}	0.0160 ± 20%	5.0×10^{-3} ± 39%	
k_{ieM}	s^{-1}	0.0160 ± 20%	5.0×10^{-3} ± 32%	

**Additional parameters for the (pro)drug transport model**[c]				
D_A	cm^2/s	4.42 x 10^{-7} ± 10%	1.44 x 10^{-7} ± 12%	Tissue diffusion coefficients for PR-104A, PR-104H and PR-104M. Reported values are the volume-averaged parameters of the extracellular diffusion coefficients D_{eN} with: $D = \varphi_e D_N$. Importantly, these D_N are different from commonly reported MCL diffusion coefficients that represent weighted averages of the free drug diffusion coefficients in intra- and extracellular compartments, based on treating tissue as homogeneous space.
D_H	cm^2/s	6.24 x 10^{-7} ± 55%	1.44 x 10^{-7} ± 12%	
D_M	cm^2/s	4.29 x 10^{-7} ± 6.8%	1.44 x 10^{-7} ± 12%	
φ_i	-	0.313 ± 28%	0.465 ± 1.5%	Intracellular volume fraction in tumors.
**Cellular PK/PD model parameters**[d]				
$AUC_{10\ H+M}$	μM.h	9.20 ± 28%[e]	7.64 ± 7.3%	AUC_{10} of intracellular PR-104H and M
$AUC_{10\ A}$	μM.h	9.67 ± 6.4%	76.9 ± 7.2%	AUC_{10} of intracellular PR-104A under anoxia.

[a] Inflow pO_2 and inflow AUCs of PR-104A, PR-104H and PR-104M were assumed to be the same in all inflowing vessels of the tumor microvascular network.

[b] CVs were obtained from simultaneous fitting of k_{metN}, k_{ieN} and k_{eiN} for PR-104A, PR-104H and PR-104M to cellular PK data, using nonlinear mixed effects modelling (NonMEM version 7, ICON Development Solutions) allowing for variability of parameter values. Residual unexplained variation (RUV) was 16.5% (HCT116) and 24% (SiHa).

[c] D_N and φ_i were determined from MCL data, with cellular PK parameters fixed at the values given in the table. This was done in several steps described below. In case of SiHa, D_H and D_A were determined first; by fitting a one-dimensional time-dependent solution of equation S10 to PR-104H MCL transport data (see Figure 6 for exemplary data) and aerobic PR-104A MCL transport data (6), respectively, with D_H or D_A as the fitted parameter and φ_i set to the literature value of 0.318 (6). The CV of D_H is relatively high because of high inter-experiment variability in D_H between data from three experiments (with a total of 12 MCL). This is unexplained but may be related to the instability of PR-104H ($t_{1/2}$ = 13.6 min) in culture medium at 37ºC. D_M was then determined by fitting the same equations to concentrations of PR-104A, PR-104H and PR-104M measured after exposure of anoxic MCL to PR-104A (= "anoxic PR-104A MCL data"; Figure 5A+D) (with values of D_H and D_A fixed at the pre-determined values and D_M and φ_i as the fitted parameters – the latter to account for variability in anoxic PR-104A metabolism between MCL. Numbers in the table represent mean ± CV of parameter values fitted to data from 14 (φ_i), 12 (D_H), 9 (D_A) and 5 (D_M) SiHa MCL. In case of HCT116/WT and HCT116/sPOR#6 the diffusion coefficients for PR-104A, PR-104H and PR-104M were assumed to be the same to limit the number of fitted parameters, which was justified by the similar diffusion coefficients for prodrug and metabolites determined in SiHa MCL. Here, anoxic PR-104A MCL data (Figure 5B+E) were initially used to fit φ_i and D_N simultaneously (since no literature value was available for φ_i). Next, D_N in aerobic MCL was determined by fitting equation S10 to aerobic PR-104A MCL transport data (Figure 5B), with D_N as the fitted

parameter and φ_i fixed at the value determined for anoxic MCL. There was a statistically significant (p = 0.004, t-test) but minor difference in the value of D_N fitted to aerobic (1.32 ± 0.020 cm^2/s; n = 3) and anoxic (n = 3; 1.57 ± 0.020 cm^2/s; n = 3) HCT116/WT MCL data. This may be related to a lower packing density of anoxic MCLs permitting higher paracellular transport as suggested by the lower apparent (urea-derived) MCL thickness and higher diffusion coefficient of the cell-excluded paracellular transport marker ^{3}H-mannitol determined for anoxic than aerobic MCLs (data not shown). D_N and φ_i estimates were slightly higher for HCT116/sPOR#6 than WT MCL, with φ_i 0.577 ± 0.044 (P = 0.143; t-test) and D_N 1.97 ± 0.10 x 10^{-7} (anoxia; n = 3; P = 0.058; t-test) or 1.64 ± 0.02 x 10^{-7} (oxia; n = 3; P < 0.001; t-test). Average values of anoxic and aerobic estimates for D_N are given in the table.

[d] Estimated from linear regression of cellular PK/PD data similar to the data shown in Figure 3D-I with the difference that AUC was derived from the concentration-time profile estimated by the cellular PK model instead from measured data. The AUC_{10} values derived by the two methods are very similar due to the good agreement of measured and model-estimated PK (Figure 3A-C).

[e] Average of the $AUC_{10\ H+M}$ values determined under anoxia (7.36 µM.h ± 3.8%) and aerobic conditions (11.0 µM.h ± 7.2%) in single cell suspensions following addition of PR-104A (Figure 3G) and PR-104H (Figure 4C+D).

Supplementary Figures

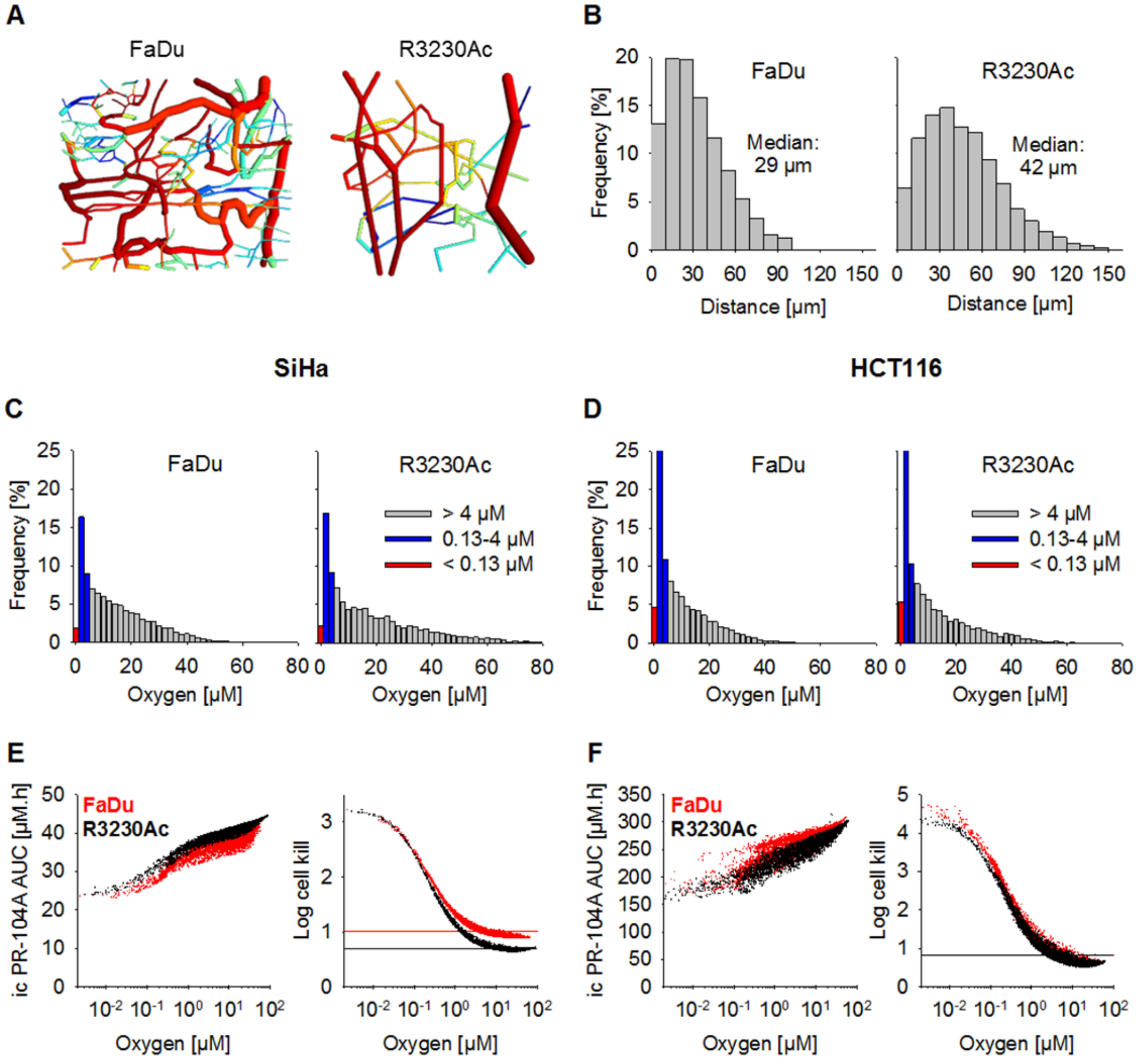

Figure S1 | Sensitivity of the SR-PK/PD model to microvascular network features. (A) Illustration of the FaDu microvascular network (990 x 810 x 150 µm) and the R3230Ac microvascular network (550 x 520 x 230 µm), generated using the program cmgui-wx-msvc. **(B)** Frequency distribution of the distances to the nearest blood vessel of tissue points in the two networks. **(C,D)** Frequency histogram of O_2 concentrations using the O_2 transport parameters for SiHa **(C)** and HCT116 **(D)**, with values of inflow pO_2 adjusted to achieve similar hypoxia in the FaDu and R3230Ac networks (see Supplementary Table 1). The red column represents the anoxic fraction (0-0.13 µM O_2). **(E,F)** SR-PK/PD model simulations for 562 µmol/kg PR-104 using the SiHa model **(E)** or the HCT116 model **(F)**. Graphs show the distribution of intracellular (ic) PR-104A AUC (left panels) and log cell kill (right panels) as a function of O_2 in the FaDu (red) and R3230Ac (black) tumor microregion. Lines represent averaged cell kill for the whole tumor microregion (overlapping for FaDu and R3234Ac in **(F)**). Note the difference in bystander killing at > ~0.1 µM O_2 for SiHa **(E)**, reflecting the lower distance between PR-104A-activating cells and better oxygenated bystander target cells at the lower intervessel distances of the FaDu network **(B)**. The lower sensitivity of bystander killing in HCT116 tumors to microvascular network features **(F)**

probably reflects the lower penetration half distance of bystander metabolites in HCT116 than SiHa tumor tissue (see section 3.1.3). The small differences in killing are accounted for by the slight differences in PR-104A AUC between the two networks caused by their different intervessel distances. At the higher PR-104A consumption rate in SiHa tumors **(E)** that causes more rapid extraction of PR-104A from the blood, differences in PR-104A AUC were dominated by the 1.8-fold difference in blood inflow (Q) per tissue volume between the 2 microvascular networks (caused by setting Q to the same value when using the R3230Ac and FaDu regions with volumes of 0.065 mm^3 and 0.12 mm^3).

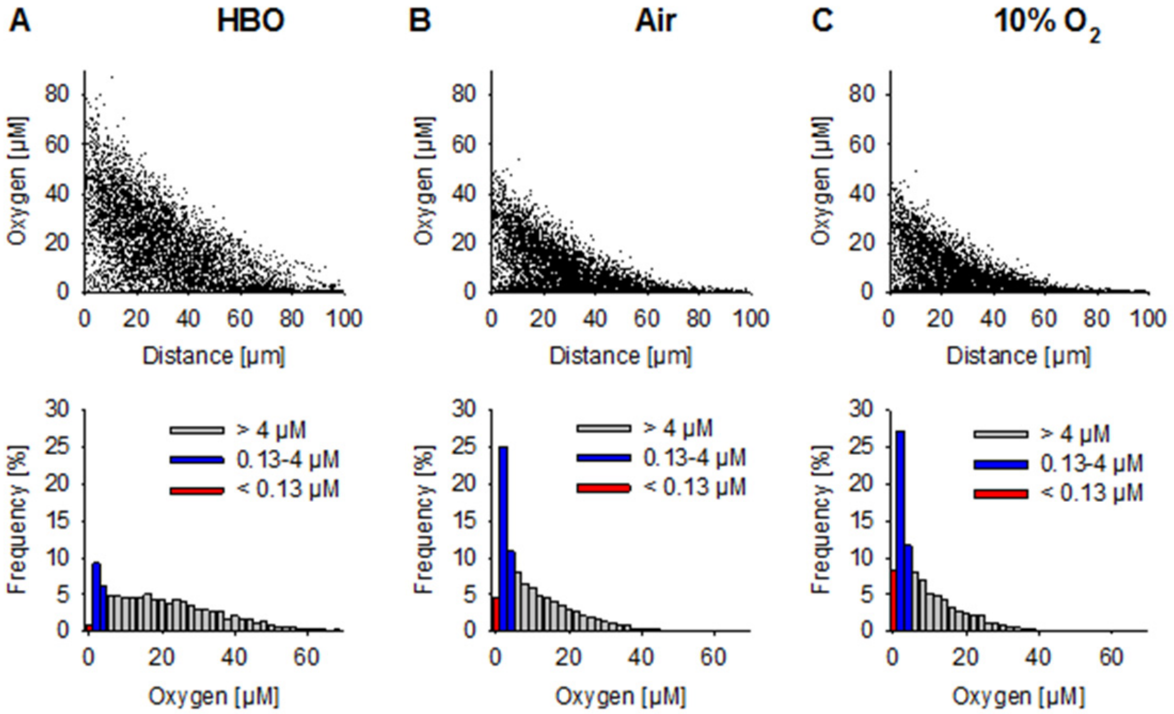

Figure S2 | Oxygenation in the virtual FaDu tumor microregion to simulate HCT116 tumors. in mice breathing hyperbaric oxygen (A) air (B) and 10% O_2 (C). Upper panels show O_2 concentration as a function of distance from the nearest blood vessel. Symbols represent 5377 small, approximately cubic regions on a grid in the FaDu tumor microregion. Lower panels show frequency histograms of O_2 concentrations. Tumor oxygenation was modeled using the O_2 transport parameters reported in Supplementary Table S1, with values of inflow O_2 concentrations at 90 µM **(A)**, 53 µM **(B)**, and 48 µM (**C**) to achieve a fraction < 1 µM O_2 in the tumor microregion similar to the mean fraction of HCT116 or SiHa tumors staining positive for the 2-nitroimidazole hypoxia probe EF5 (Figure 13F) under the different oxygen conditions.

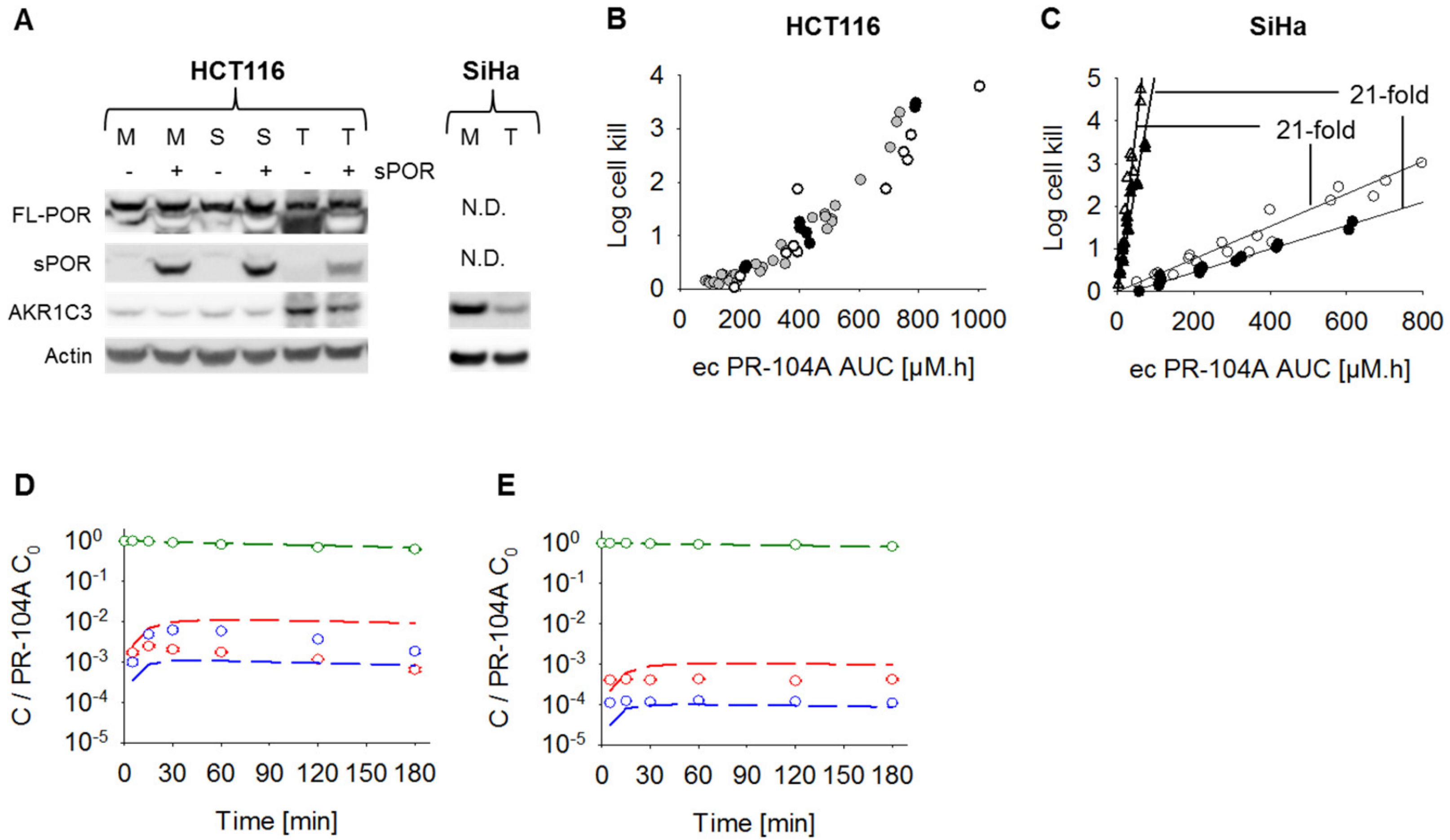

Figure S3 | Comparison of PR-104A reductase expression and cellular PK/PD *in vitro* and *in vivo*. (A) Western blot analysis of POR and AKR1C3 expression in SiHa, HCT116/WT (-sPOR) and HCT116/sPOR#6 (+sPOR) cells grown as monolayers (M), multicellular spheroids (S) or individual tumor xenografts (T). Cell lysates were prepared in SDS buffer and ~20 µg of protein was loaded on SDS-PAGE gels (NuPAGE 4-12% Bis-Tris gel; Invitrogen, USA), transferred, blocked, probed with primary mouse monoclonal antibodies against POR (10), AKR1C3 and Actin (11) as reported, and incubated with HRP-coupled goat anti-mouse secondary antibody (sc-2055, Santa Cruz Biotechnology Inc., USA; 1:10000 dilution). Actin, sPOR and AKR1C3 in SiHa samples were visualized using SuperSignal West Pico Chemiluminescent Substrate

(Thermo Scientific, USA), while endogeneous full-length POR (FL-POR) and AKR1C3 in HCT116 samples were visualized with the more sensitive ECL Advance Western Blotting Detection Kit (GE Healthcare). FL-POR (76.9 kDa) was well resolved from sPOR (70 kDa), which represents the lower, overexposed band in row 1. AKR1C3 expression in SiHa appeared to be lower *in vivo* than *in vitro*, which might be due to the presence of murine cells *in vivo* that do not express the human enzyme AKR1C3, but do express actin that is recognized by the pan-actin antibody used in this experiment (12). In case of HCT116 cell lines the AKR1C3:Actin ratio was ~1.7-fold higher *in vivo* than *in vitro*. N.D. not determined. Despite these differences in AKR1C3 expression, the aerobic PR-104A PK/PD relationships were similar *in vitro* and *ex vivo* **(B,C)** suggesting that PR-104A metabolism at 20% O_2 may be dominated by residual one-electron reduction (due to incomplete inhibition by O_2) rather than AKR1C3. Graphs **B+C** show the relationship between log cell kill and extracellular (ec) PR-104A AUC measured in suspensions of HCT116 **(B)** and SiHa **(C)** cells derived from tumor xenografts (black) or from *in vitro* culture (white + grey; SiHa from monolayer, HCT116 from spheroid culture) following addition of PR-104A under aerobic (circles) or anoxic (triangles) conditions. Symbols represent surviving fractions in individual vials at different time points while lines show linear regression fits. **(B)** ~2 x 10^6 cells/ml; initial PR-104A concentration (C_0): 15 and 30 μM (anoxic), 100 and 200 μM (aerobic) with duplicate vials of cells for each C_0. **(C)** black + white: 1 x 10^6 cells/ml, C_0: 400 and 800 μM with duplicate vials for each C_0; grey: 2 x 10^6 cells/ml (data from the experiment reported in Figure 3B,E,H). The non-linear PR-104A PK/PD relationship suggests a second mechanism of PR-104A-mediated cell kill in addition to DNA interstrand cross-linking (ICL) by PR-104H+M (possibly due to redox cycling, i.e. the generation of reactive oxygen species resulting from re-oxidation of the PR-104A nitro radical by oxygen). This ICL-independent mechanism was not considered in the SR-PK/PD model because it only contributed at PR-104A AUC > 400 μM.h, a value much higher than the free PR-104A plasma AUC in mice after administration of 562 μmol/kg PR-104 (Supplementary Table S1). **(D,E)** Concentrations of extracellular PR-104A (green), PR-104H (red) and PR-104M (blue) in anoxic **(D)** and aerobic **(E)** suspensions of SiHa cells derived from tumor xenografts. Symbols represent mean ± SEM of concentrations (normalized to C_0) for 4 replicate vials and lines show predictions of the cellular PK model for SiHa (Supplementary table S1). The good agreement of model-estimates (based on parameters determined in SiHa cell suspensions derived from monolayer culture, Figure 3A) and measurements for total reduced metabolites suggests that there are no major differences in the PK parameters in cells from tumor xenografts, although the different relative contributions of PR-104H and PR-104M to total reduced metabolites under anoxia **(D)** may reflect a different expression profile of PR-104H- and PR-104M-metabolizing enzymes in tumors. This is not expected to substantially alter PR-104 antitumor activity if PR-104H and PR-104M are approximately equitoxic and have similar tissue transport properties.

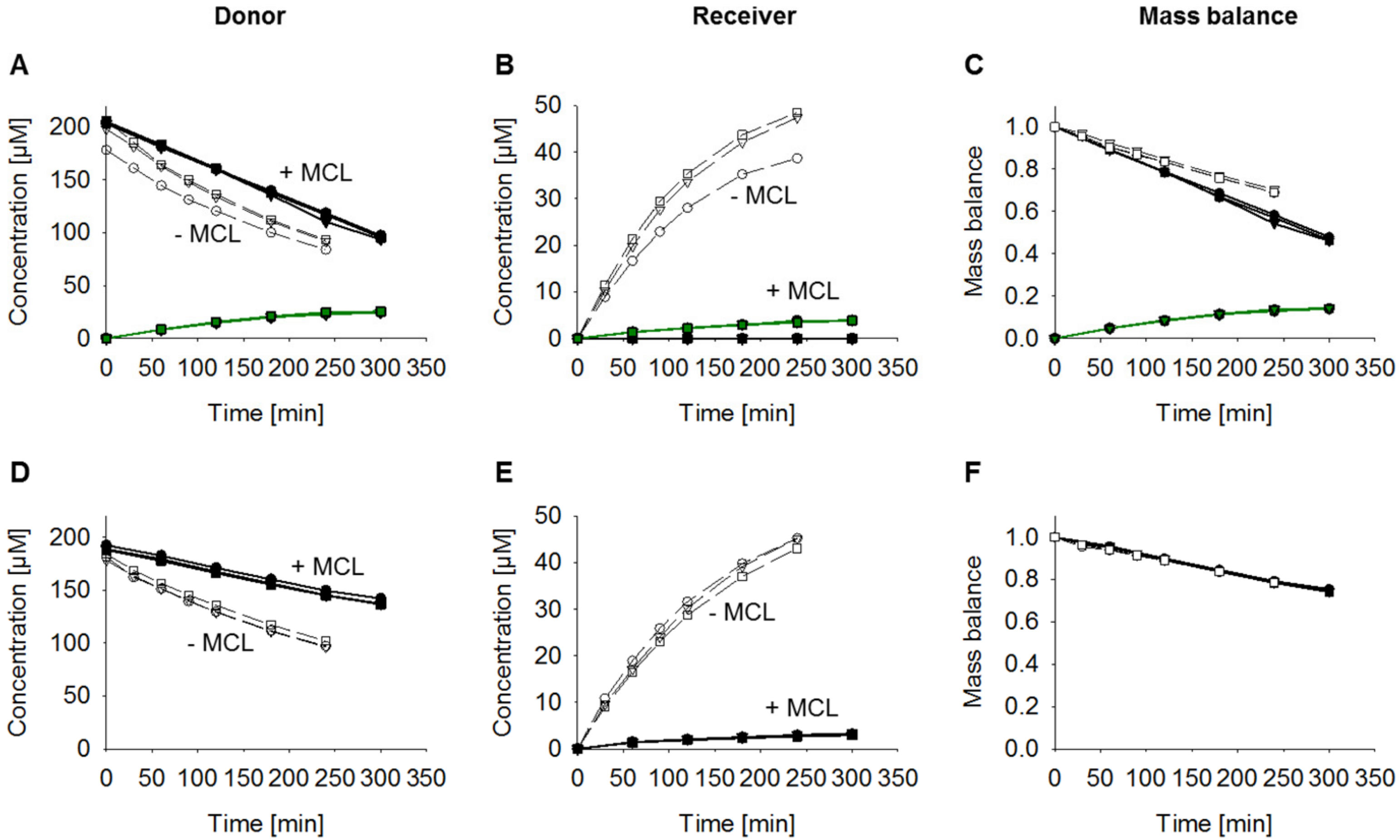

Figure S4. Transport of PR-104 (**A-C**) and the *O-β*-glucuronide of PR-104A (PR-104G, synthesized as described (13); **D-E**) through support membranes (open symbols, dashed lines) and SiHa MCL (closed symbols, solid lines) under oxic conditions following their addition to the donor compartment at initial concentrations of 200 µM. Graphs show concentration-time profiles of the respective compounds and of PR-104A (green) in the donor (**A+D**) and receiver compartments (**B+E**), as well as mass balance (**C+F**). Different symbols and lines represent separate MCL with comparable thickness as assessed by transport of ^{14}C-urea internal standard. PR-104, PR-104A and PR-104G were quantified by LC-MS as described previously (14), with mass transitions for PR-104G as reported in (15). PR-104 could not be detected in receiver compartments in the presence of MCL (**B**).

References

1. Gray LH, Steadman JM. Determination of the oxyhaemoglobin dissociation curves for mouse and rat blood. J Physiol (1964) **175**: 161-71.
2. Pries AR, Cornelissen AJ, Sloot AA, Hinkeldey M, Dreher MR, Hoepfner M, et al. Structural adaptation and heterogeneity of normal and tumor microvascular networks. PLoS Comput Biol (2009) **5**. doi: 10.1371/journal.pcbi.1000394
3. Secomb TW, Hsu R, Park EY, Dewhirst MW. Green's function methods for analysis of oxygen delivery to tissue by microvascular networks. Ann Biomed Eng (2004) **32**: 1519-29.
4. Dewhirst MW, Secomb TW, Ong ET, Hsu R, Gross JF. Determination of local oxygen consumption rates in tumors. Cancer Res (1994) **54**: 3333-6.
5. Secomb TW, Hsu R, Braun RD, Ross JR, Gross JF, Dewhirst MW. Theoretical simulation of oxygen transport to tumors by three-dimensional networks of microvessels. Adv Exp Med Biol (1998) **454**: 629-34.
6. Hicks KO, Myint H, Patterson AV, Pruijn FB, Siim BG, Patel K, et al. Oxygen dependence and extravascular transport of hypoxia-activated prodrugs: comparison of the dinitrobenzamide mustard PR-104A and tirapazamine. Int J Radiat Oncol Biol Phys (2007) **69**: 560-71.
7. Williams MV, Denekamp J, Fowler JF. A review of alpha/beta ratios for experimental tumors: implications for clinical studies of altered fractionation. Int J Radiat Oncol Biol Phys (1985) **11**: 87-96.
8. Carlson DJ, Keall PJ, Loo BW, Jr., Chen ZJ, Brown JM. Hypofractionation results in reduced tumor cell kill compared to conventional fractionation for tumors with regions of hypoxia. Int J Radiat Oncol Biol Phys (2011) **79**: 1188-95.
9. Patel K, Choy SF, Hicks KO, Melink TJ, Holford NHG, Wilson WR. A combined pharmacokinetic model for the hypoxia-targeted prodrug PR-104A in humans, dogs, rats and mice predicts species differences in clearance and toxicity. Cancer Chemother Pharmacol (2011) **67**: 1145-55.
10. Guise CP, Abbattista MR, Tipparaju SR, Lambie NK, Su J, Li D, et al. Diflavin oxidoreductases activate the bioreductive prodrug PR-104A under hypoxia. Mol Pharmacol (2012) **81**: 31-40.
11. Guise CP, Abbattista M, Singleton RS, Holford SD, Connolly J, Dachs GU, et al. The bioreductive prodrug PR-104A is activated under aerobic conditions by human aldo-keto reductase 1C3. Cancer Res (2010) **70**: 1573-84.
12. Lessard JL. Two monoclonal antibodies to actin: one muscle selective and one generally reactive. Cell Motil Cytoskeleton (1988) **10**: 349-62.
13. Gu Y, Atwell GJ, Wilson WR. Metabolism and excretion of the novel bioreductive prodrug PR-104 in mice, rats, dogs and humans. Drug Metab Dispos (2010) **38**: 498-508.
14. Patel K, Lewiston D, Gu Y, Hicks KO, Wilson WR. Analysis of the hypoxia-activated dinitrobenzamide mustard phosphate prodrug PR-104 and its alcohol metabolite PR-104A in plasma and tissues by liquid chromatography-mass spectrometry. J Chromatogr B Analyt Technol Biomed Life Sci (2007) **856**: 302-11.
15. Gu Y, Wilson WR. Rapid and sensitive ultra-high-pressure liquid chromatography-tandem mass spectrometry analysis of the novel anticancer agent PR-104 and its major metabolites in human plasma: Application to a pharmacokinetic study. J Chromatogr B Analyt Technol Biomed Life Sci (2009) **877**: 3181-6.
